# Unraveling the role of prenyl side-chain interactions in stabilizing the secondary carbocation in the biosynthesis of variexenol B

**DOI:** 10.3762/bjoc.19.107

**Published:** 2023-09-28

**Authors:** Moe Nakano, Rintaro Gemma, Hajime Sato

**Affiliations:** 1 Interdisciplinary Graduate School of Medicine and Engineering, University of Yamanashi, 4-4-37 Takeda, Kofu, Yamanashi 400-8510, Japan; 2 PRESTO, Japan Science and Technology Agency, Kawaguchi, Saitama 332–0012, Japan

**Keywords:** biosynthesis, carbocation, cation–π interaction, DFT, terpene

## Abstract

Terpene cyclization reactions involve a number of carbocation intermediates. In some cases, these carbocations are stabilized by through-space interactions with π orbitals. Several terpene/terpenoids, such as sativene, santalene, bergamotene, ophiobolin and mangicol, possess prenyl side chains that do not participate in the cyclization reaction. The role of these prenyl side chains has been partially investigated, but remains elusive in the cyclization cascade. In this study, we focus on variexenol B that is synthesized from iso-GGPP, as recently reported by Dickschat and co-workers, and investigate the possibility of through-space interactions with prenyl side chains using DFT calculations. Our calculations show that (i) the unstable secondary carbocation is stabilized by the cation–π interaction from prenyl side chains, thereby lowering the activation energy, (ii) the four-membered ring formation is completed through bridging from the exomethylene group, and (iii) the annulation from the exomethylene group proceeds in a barrier-free manner.

## Introduction

Terpene/terpenoids are most abundant natural products in nature, more than 180,000 terpenoid compounds have been reported to date [1–4]. One of the most intriguing point is that all diversified structures are synthesized from common starting materials, isoprenoids. Reactions that generate complex cyclic structures and multiple stereocenters from linear achiral precursors offer many valuable insights from a fundamental organic chemistry perspective.

The terpene cyclization cascade generally involves a multistep domino-type reaction. Therefore, it is challenging to reveal the detailed reaction mechanism solely by an experimental method. To address this issue, computational chemistry including DFT [5–9], QM/MM [10–16] and QM/MM MD [14–17] calculations have been used for the biosynthetic studies of terpene/terpenoids [18].

Terpene-forming reactions, which involve various types of carbocation species stabilized by hyperconjugative interactions, through-space interactions, and C–H–π interactions, have been intensively investigated by Tantillo and co-workers, who have contributed greatly to revealing the intriguing nature of carbocations [7,19–20].

We have also elucidated various new insights of carbocation chemistry, such as the C–H–π interaction between the carbocation intermediate and the Phe residue of terpene cyclase in the biosynthesis of sesterfisherol [21], and the intricated rearrangement reaction mechanism promoted by the equilibrium state of the homoallyl cation and the cyclopropylcarbinyl cation in the biosynthesis of trichobrasilenol [22], by combined methods of computational and experimental chemistry.

**Scheme 1:**
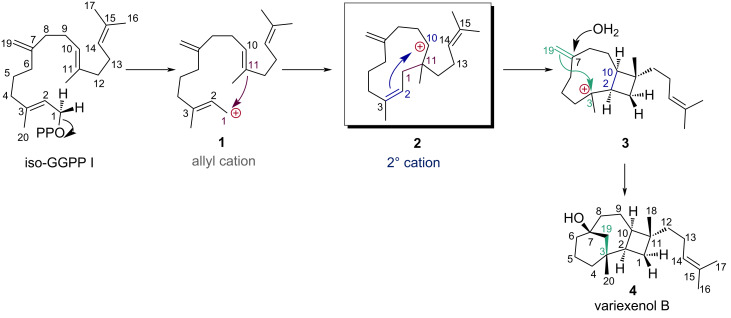
Proposed biosynthetic pathway for variexenol B.

Recently, Dickschat et al. reported the synthesis of a novel diterpene compound, variexenol B, using a substrate analogue called iso-GGPP (Scheme 1) [23]. This biosynthetic pathway has two interesting aspects. First, this cyclization cascade involves a prenyl side chain that do not participate in the cyclization cascade. This type of terpene compounds has already been reported, such as santalene, bergamotene, mangicol, etc. The idea that the reaction mechanism changes due to differences in the prenyl side chains has been studied by Tantillo and co-workers [24–26]. They reported that the carbocation intermediates traversed in pinene/camphene and ylangene/sativene biosynthesis change depending on the presence or absence of prenyl side chains. In their study, it was argued that the extent of hyperconjugation determines whether the reaction proceeds in a stepwise or concerted manner.

The second interesting aspect of the biosynthesis of variexenol B is that the biosynthetic pathway involves an intermediate with an exomethylene group. A terpene with an exomethylene group as a starting material is rare. Several terpene cyclizations with an exomethylene group are known, such as with caryolene and crotinsulidane diterpenoids, and the reaction mechanisms have been analyzed [27–30]. It would be interesting to see how the exomethylene group reacts in the cyclization of variexenol B. In this study, we investigated the biosynthetic pathways using DFT calculations to validate the above-mentioned aspects.

## Results and Discussion

**Figure 1:**
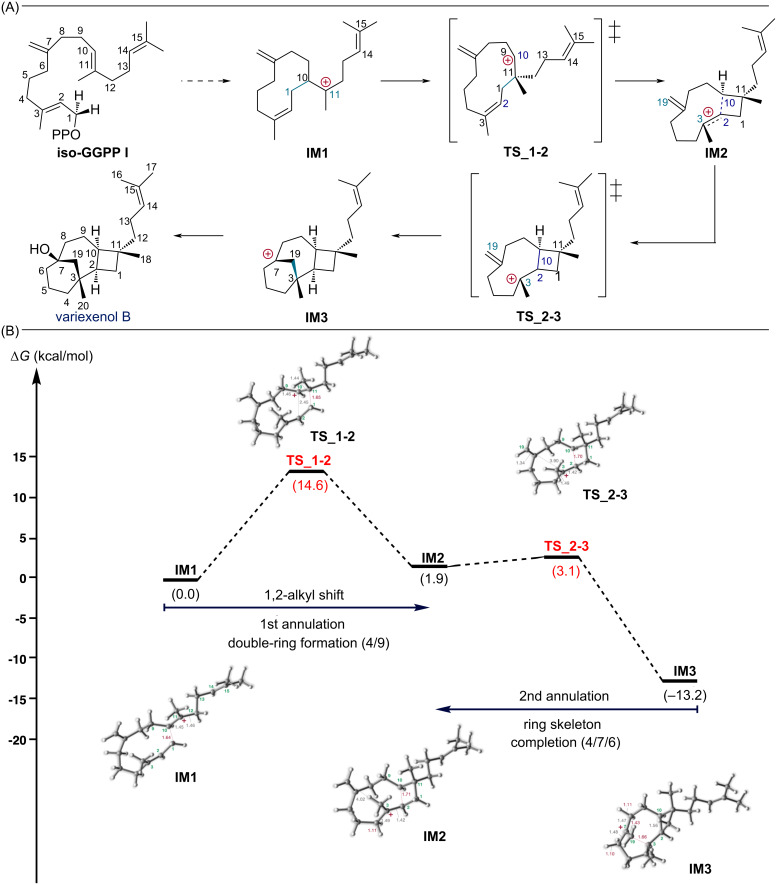
(A) Results of DFT evaluation of the whole pathway of variexenol B without cation–π interaction. (B) Energy diagram of variexenol B without cation–π interaction. IM means intermediate and TS transition state. Potential energies (kcal/mol, Gibbs free energies calculated at the mPW1PW91/6-31+G(d,p)//M06-2X/6-31+G(d,p) level) relative to **IM1** are shown in parentheses.

**Figure 2:**
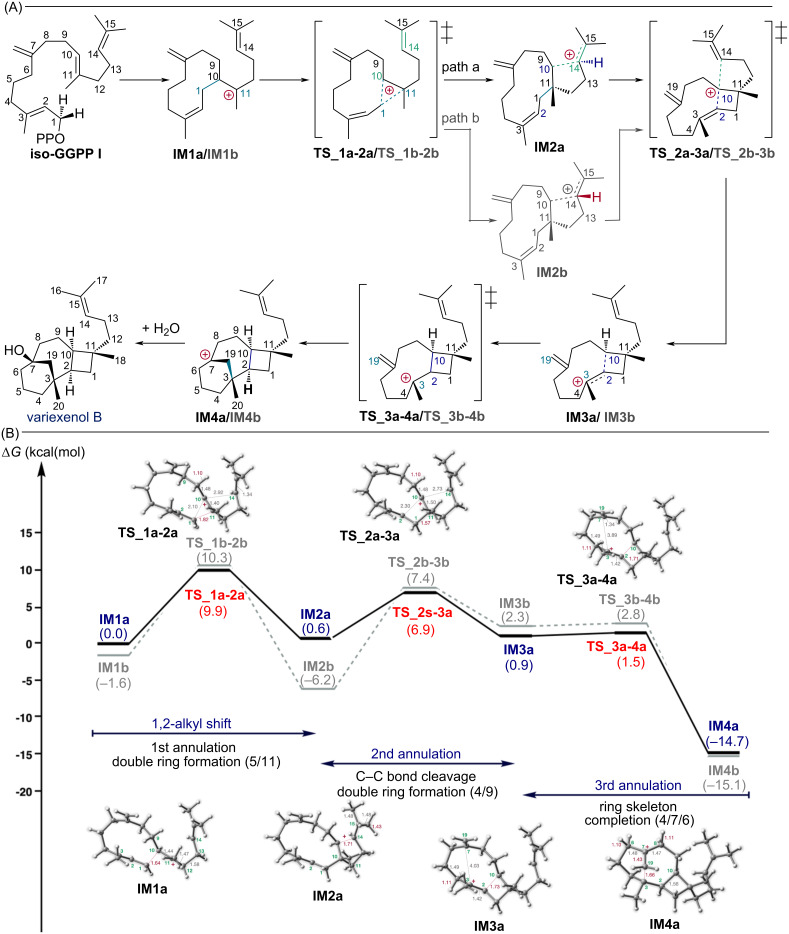
(A) Results of the DFT evaluation of the whole pathway of variexenol B including cation–π interaction from the prenyl side chain. Path a has an α-hydrogen at the C14 position in **IM2**, while path b has the opposite orientation. (B) Energy diagram of variexenol B with consideration of cation–π interaction. Potential energies (kcal/mol, Gibbs free energies calculated at the mPW1PW91/6-31+G(d,p)//M06-2X/6-31+G(d,p) level) relative to **IM1** are shown in parentheses.

The detailed structures of the intermediates and transition states were elucidated by computational analysis. Interestingly, we have found an interaction between the secondary carbocation and the prenyl side chain. Figure 1 shows the computed biosynthetic pathway and energy diagram without cation–π interaction, while Figure 2 shows the computed biosynthetic pathway including cation–π interaction from the prenyl side chain.

Our research started with the application of DFT calculations to the putative biosynthetic pathway of variexenol B (Figure 1). It was revealed that the variexenol B biosynthetic pathway undergoes a two-step reaction process. Contrary to the putative biosynthetic pathway, the formation of the C1–C11 and C2–C10 bonds was found to be concerted, due to the formation of a secondary carbocation at the C10 position. Then, the tertiary carbocation formed at the C3 position undergoes virtually barrier-free cyclization from the exomethylene group to yield **IM3**.

We next investigated the effect of the prenyl side chain in the biosynthesis of variexenol B. Although several terpene compounds with prenyl side chains have been reported, it remains unclear whether these prenyl side chains are located inside or outside the active site during the cyclization process. Therefore, we searched for conformations in which the side chain is closer to the carbocation center and performed calculations.

It was found that the structure with the prenyl side chain containing the C14=C15 double bond positioned inwards was more advantageous than the pathway shown in Figure 1. Calculations based on the specified structure are shown in Figure 2. In this pathway, the C14=C15 double bond interacts with the secondary carbocation at C10, reducing the activation energy of the first step by approximately 4.7 kcal/mol. Moreover, due to the stabilization of the secondary carbocation-like intermediate **IM2**, the reaction proceeds stepwise rather than concertedly [7]. It was found that the final cyclization reaction from the exomethylene group proceeds without a barrier, similar to the previous pathway.

Regarding the orientation of the prenyl side chain, two pathways can be considered depending on whether the hydrogen at C14 is pointing; α-hydrogen (path a) or β-hydrogen (path b). Both pathways follow similar reaction mechanisms, however, when comparing path a and path b, the most striking energy difference is in the step from **IM2a/b** to **IM3a/b** (Figure 2B). The energy barrier of this step is 6.3 kcal/mol for path a, whereas 13.6 kcal/mol for path b, with a difference of 7.3 kcal/mol. Although the stabilization of the intermediate **IM2b** is greater in path b, the activation energy suggests that path a is more favorable.

Generally, the activation energies for terpene cyclization reactions are often below 10 kcal/mol. However, in the case of complex rearrangement reactions involving secondary carbocations, which we recently discovered, reactions with activation energies around 16 kcal/mol have been reported [22]. In the pathway shown in Figure 1, the highest energy barrier was 14.6 kcal/mol. Conversely, in Figure 2, path a had an energy barrier of 9.9 kcal/mol and path b 13.6 kcal/mol. From these results, it can be concluded that although all three pathways have the potential to advance the reaction, the most energetically favorable pathway is path a, as shown in this study.

To the best of our knowledge, the interaction from the prenyl side chain towards the carbocation center have not been reported. Systems with secondary carbocations on rings bearing prenyl side chains are commonly observed in steroid biosynthesis. These type of cyclization reactions have been vigorously studied by Hess [31–36] and Wu [37–38]. In these systems, the secondary carbocation and the double bond of the neighboring prenyl side chain interacts and promptly induce C–C bond formation. There have been no reports published where, as in our case, the cation is stabilized without bond formation. We have also considered other transannular cation–π interactions in this system. In this case, the interaction between the secondary carbocation at C10 and the C2=C3 double bond or the exomethylene group at C7 should be considered. However, moving it closer to the C2=C3 bond would result in **IM2** as shown in Figure 1 and a C–C bond would be formed. The exomethylene group at C7 is also very reactive, so if it gets close, it would easily form a C–C bond. Therefore, we believe that no other transannular cation–π interactions need to be considered in this system.

In systems without cation–π interactions, such as in the biosynthesis of variediene [39] and spiroviolene [40], bonds around the secondary carbocation are strongly influenced by hyperconjugation. In particular, C–C bonds containing a secondary cation are shortened to about 1.45 Å, showing a slight double bond character. On the other hand, in intermediates such as **IM2a** and **IM2b**, which have cation–π interactions, the surrounding bonds are hardly affected by hyperconjugation (C9–C10: 1.54 Å, C10–C11: 1.54 Å, C11–C1: 1.57 Å). We have also done a comparative analysis of the charge distribution in scenarios with and without cation–π interactions. In cases where the interaction is absent, the cationic character at C10 is pronounced. Conversely, in the presence of the cation–π interaction, the cation is delocalized, resulting in a decrease in cationicity at C10 and a corresponding increase in cationicity at C15.

Note that the interconversion of **TS_2a-3a** to **TS_2b-3b** requires a significant conformational change, such as a 180 degree rotation of the iPr group. However, such a large conformational change is unlikely to occur within the enzyme. Therefore, the Curtin–Hammett principle is not applicable to this system.

**Figure 3:**
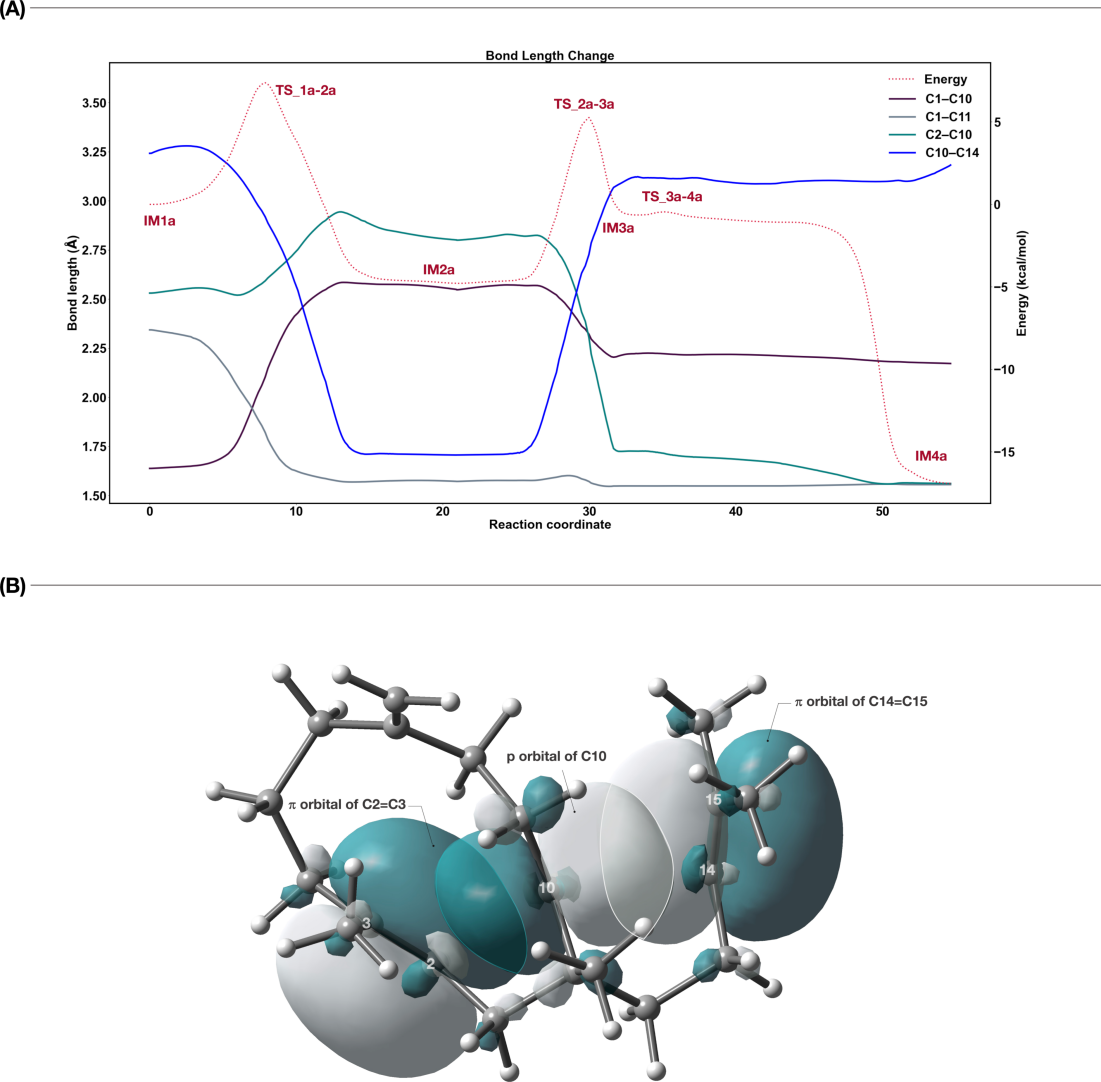
(A) A representative example of the evolution of key bond lengths in the conversion of path a. (B) Key representative orbitals of **TS_2a–3a** computed by DFT calculations.

To investigate the details of carbocations and hyperconjugations in the variexenol B biosynthetic pathway, we carried out a bond length change analysis on the bonds that contribute most to the reaction from **IM1a** to **IM4a** (Figure 3A).

In the process from **IM1a** to **TS_1a–2a**, the C1–C10 bond ruptures as C1 shifts towards C11. Subsequently, in the transition towards **IM2a**, a complete formation of the C1–C11 bond occurs. At this point, the vacant orbital of the carbocation at C10 interacts with the π orbital of the C14=C15 double bond.

The distance between C10 and C14 is 1.71 Å, which is hardly to recognize as a single C–C bond, since the distance is greatly elongated. Moreover, the bond length of C14=15 is 1.43 Å, which is close to the double bond length. Judging from the bond length alone, it is not impossible to conclude that the C10–C14 bond is formed, but considering the rational mechanism of organic reactions, bond cleavage does not occur immediately after the bond is formed.

On the other hand, the C10–C14 bond length of **IM2b** is 1.64 Å, which is the bond length when hyperconjugated and is commonly observed in terpene-forming reactions. This relatively short bond length appears to contribute to the stability of **IM2b**. The energy difference between **IM2a** and **IM2b** appears to be due to small conformational differences caused by the stereochemistry of H14.

In **TS_2a–3a**, the C10 secondary carbocation is stabilized and sandwiched between the two π orbitals of C2=C3 and C14=C15. The status of this orbital interaction is depicted in Figure 3B. This interaction forms the C2–C10 bond and the reaction proceeds to **IM3a**.

Regarding the 4-membered ring formation, the C2–C10 bond in **IM3a** is 1.73 Å long, which is hard to recognize as a single bond. However, it is well consistent with the previously reported hyperconjugation in 4-membered ring formation [7]. Then, the C2–C10 bond became 1.56 Å and the 4-membered ring bond is completed (Figure 3B) when the hyperconjugation effect is eliminated by the removal of the C3 carbocation through annulation from the exomethylene group. Based on the key bond analysis, we have successfully elucidated the details of variexenol B biosynthesis. Note that there is an interaction between the empty p orbital of C10 and the π orbital of C14=C15 in Figure 3B, although the bond is not shown. The presence or absence of a bond in the GaussView depends only on the distance between the atoms and may differ from the actual bonding. If a bond is stretched due to hyperconjugation etc., it often happens that the bond is not displayed correctly. Therefore, we performed bond length analysis and NBO analysis to understand the state of the carbocation and bonding.

## Conclusion

In conclusion, we have investigated the detailed reaction mechanism of the biosynthesis of variexenol. We have revealed three new insights: (i) the possibility of stabilization of the secondary carbocation by the prenyl side chain of the intermediate, (ii) the four-membered ring formation is completed by the bridging reaction, and (iii) the annulation from the exomethylene group is a barrier-free process.

To date, when constructing the computational model, we have sometimes truncated the prenyl side chains that do not participate in the cyclization cascade in order to reduce the computational cost [35,41]. However, as demonstrated in this study, the possibility of cation–π interactions lowering the activation energy of annulation requires caution when constructing computational models in the future. Furthermore, future research is expected to determine whether there is space in the enzyme active site for these prenyl side chains to fold and approach the reaction center, as seen in X-ray crystallographic analysis.

## Experimental

All calculations were carried out using the Gaussian 16 package [42]. Structure optimizations were done with the M06-2X [43] density functional theory method and the 6-31+G(d,p) basis set without any symmetry restrictions. M06-2X was selected because of its accuracy in calculating terpene-forming reactions and its proven track record being used in previous studies of reaction mechanism analysis [16,44]. Vibrational frequency calculations at the same level of theory with optimization were performed to verify that each local minimum has no imaginary frequency and that each **TS** has only a single imaginary frequency. Conformational search was done with conflex program [45–47]. Intrinsic reaction coordinate (IRC) calculations [48–51] for all **TS**s were performed with GRRM11 [52] based on Gaussian 16. Single-point energies were calculated at the mPW1PW91/6-31+G(d,p) level based on the optimized structure by using the M06-2X method. The utility of relative Gibbs free energies (G*_rel_*) based on single-point energy at the mPW1PW91 level has been previously validated for a wide variety of terpene-forming reactions [22,41,53].

## Supporting Information

File 1 IRC plot, 3D representations of all computed structures, cartesian coordinates, energies, and imaginary frequencies.
