# Supplementary material for: Unraveling the role of prenyl side-chain interactions in stabilizing the secondary carbocation in the biosynthesis of variexenol B
Source: Beilstein J Org Chem. 2023 Sep 28;19:1503–10. doi: 10.3762/bjoc.19.107 (PMC10548252; doi:10.3762/bjoc.19.107)
Supplement: File 1 — IRC plot, 3D representations of all computed structures, cartesian coordinates, energies, and imaginary frequencies. [file Beilstein_J_Org_Chem-19-1503-s001.pdf]

BEILSTEIN JOURNAL OF ORGANIC CHEMISTRY

Supporting Information

for

Unraveling the role of prenyl side-chain interactions in stabilizing the secondary carbocation in the biosynthesis of variexenol B

Moe Nakano, Rintaro Gemma and Hajime Sato

Beilstein J. Org. Chem. **2023,** *19,* 1503–1510. doi:10.3762/bjoc.19.107

IRC plot, 3D representations of all computed structures, cartesian coordinates, energies, and imaginary frequencies

License and Terms: This is a supporting information file under the terms of the Creative Commons Attribution License (https://creativecommons.org/licenses/by/4.0). Please note that the reuse, redistribution and reproduction in particular requires that the author(s) and source are credited and that individual graphics may be subject to special legal provisions.
The license is subject to the *Beilstein Journal of Organic Chemistry* terms and conditions: (https://www.beilstein-journals.org/bjoc/terms)

Contents

1. IRC Plot ……………………………………………………………………………S1
2. 3D representation of all computed structures………………………………………S5
3. Cartesian coordinates, energies, and imaginary frequencies……………………….S8

1. IRC Plot

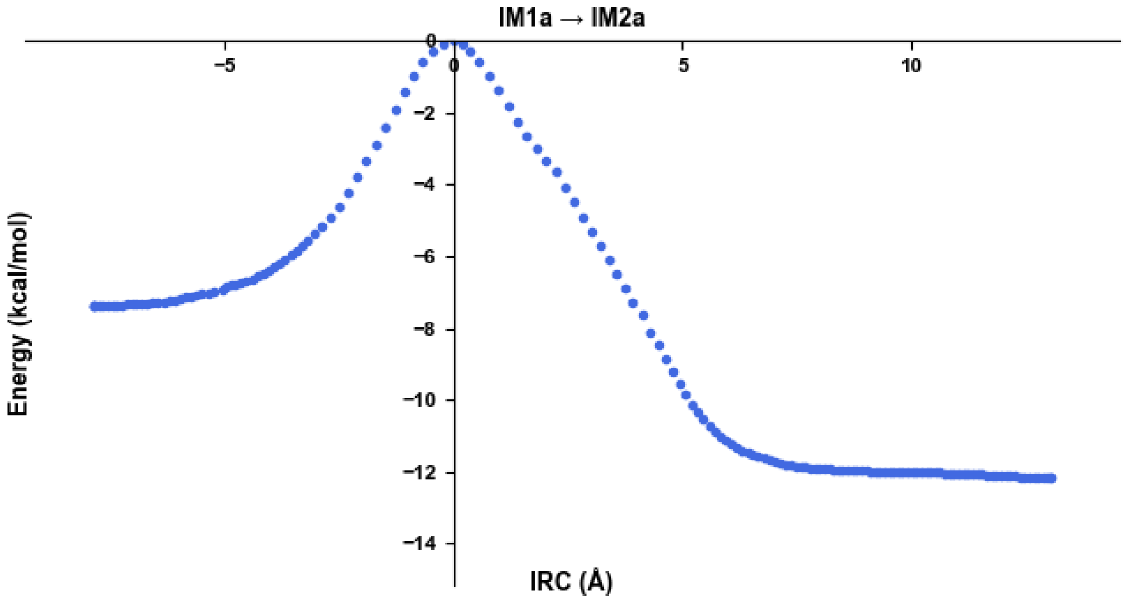
IM1a → IM2a
−5
0
5
10
0
−2
−4
−6
−8
−10
−12
−14
Energy (kcal/mol)
IRC (Å)

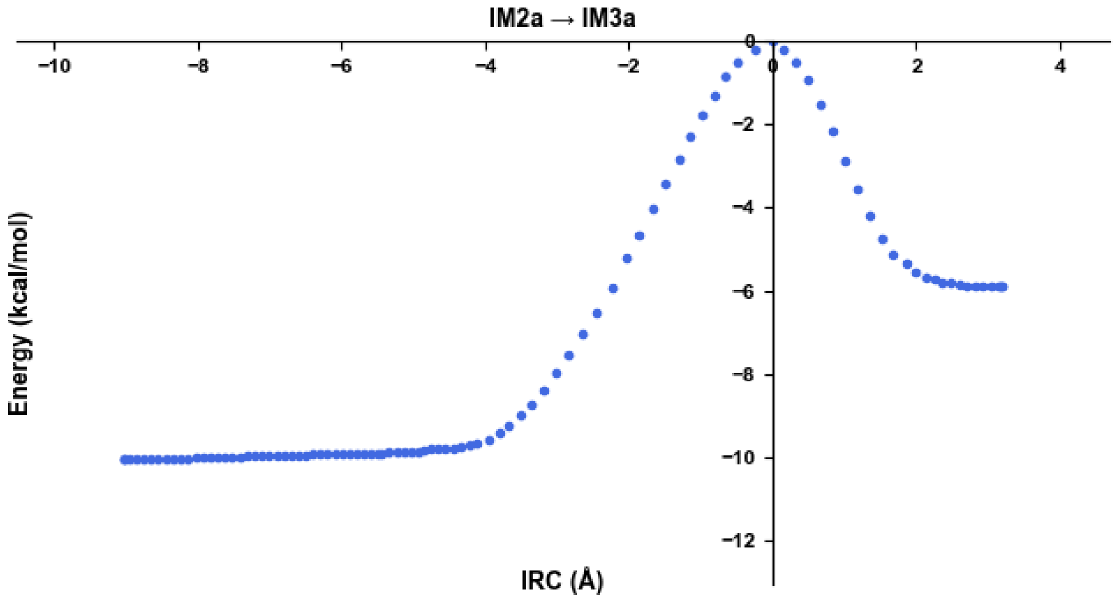
IM2a → IM3a
−10
−8
−6
−4
−2
0
2
4
0
−2
−4
−6
−8
−10
−12
Energy (kcal/mol)
IRC (Å)

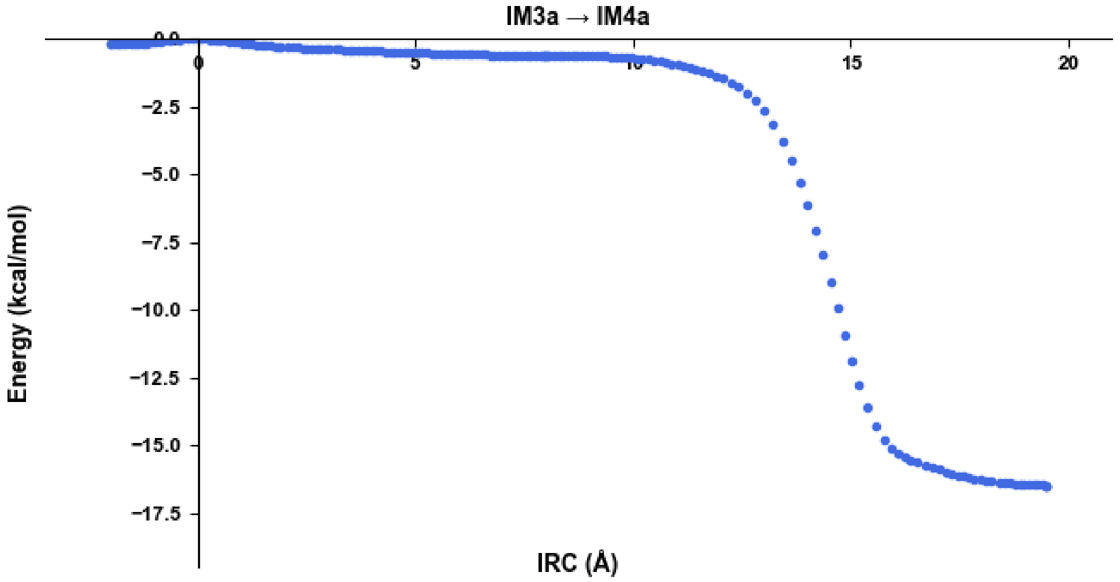
IM3a → IM4a
Energy (kcal/mol)
IRC (Å)
0.0
−2.5
−5.0
−7.5
−10.0
−12.5
−15.0
−17.5
0
5
10
15
20

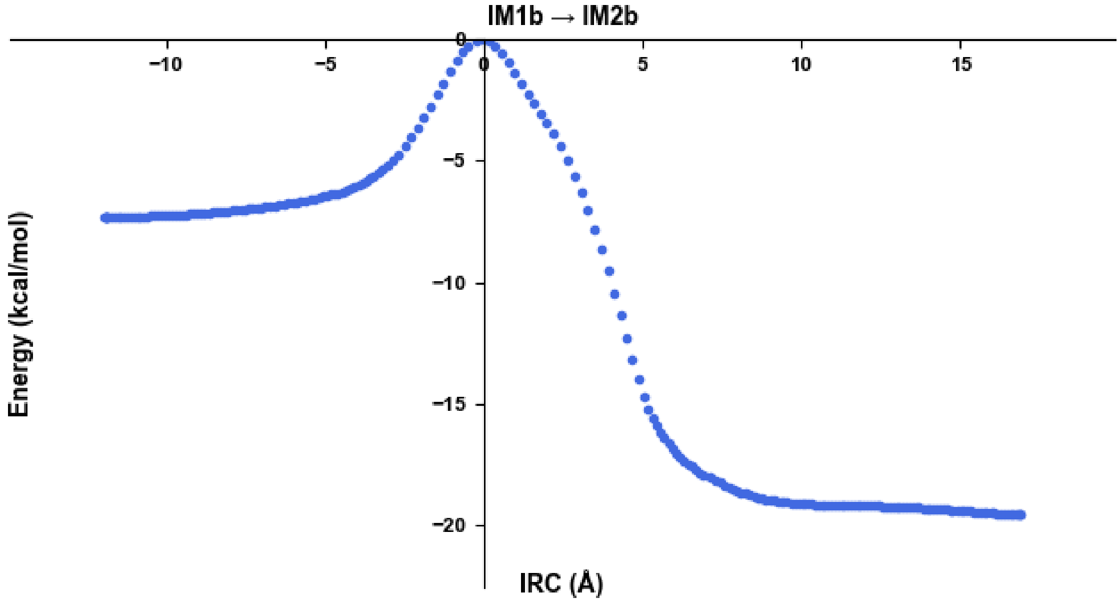
IM1b → IM2b
Energy (kcal/mol)
IRC (Å)
0
−5
−10
−15
−20
−10
−5
0
5
10
15

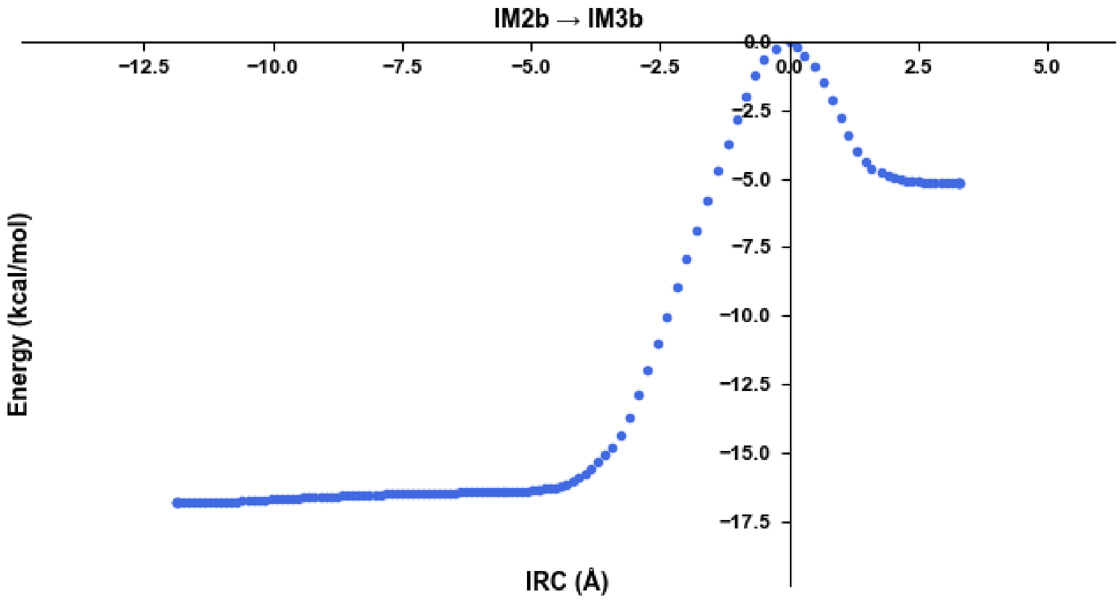

IM2b → IM3b
Energy (kcal/mol)
IRC (Å)

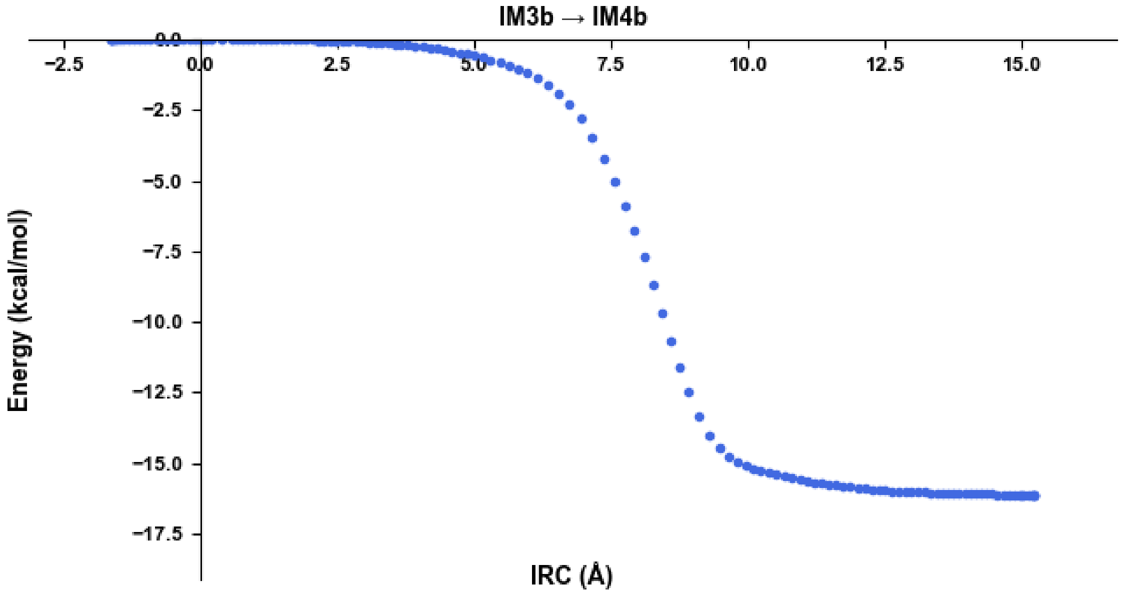

IM3b → IM4b
Energy (kcal/mol)
IRC (Å)

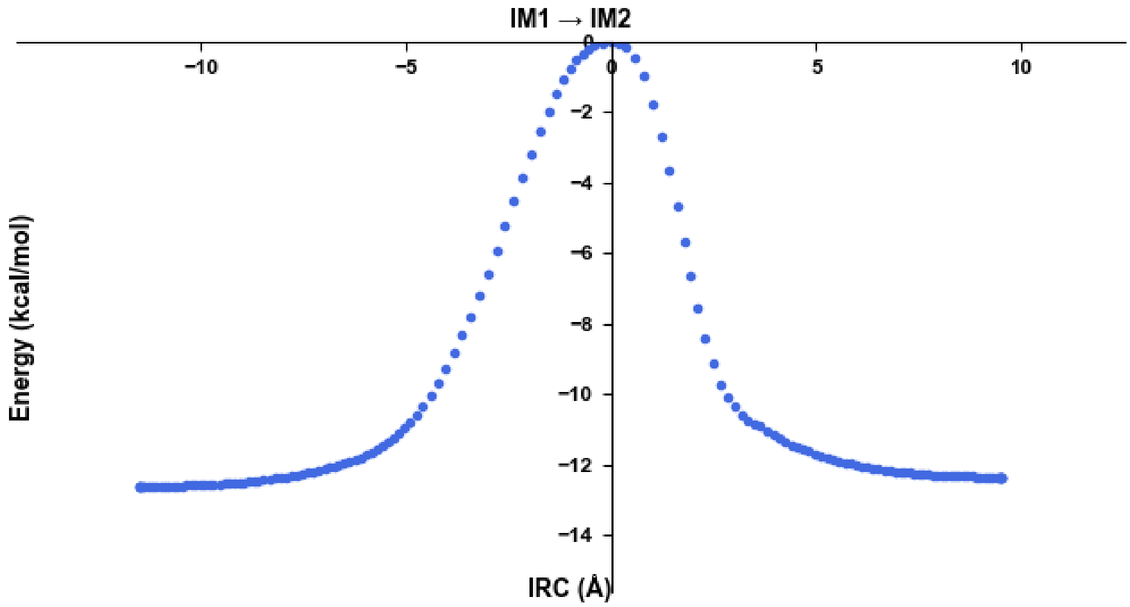

IM1 → IM2
Energy (kcal/mol)
IRC (Å)

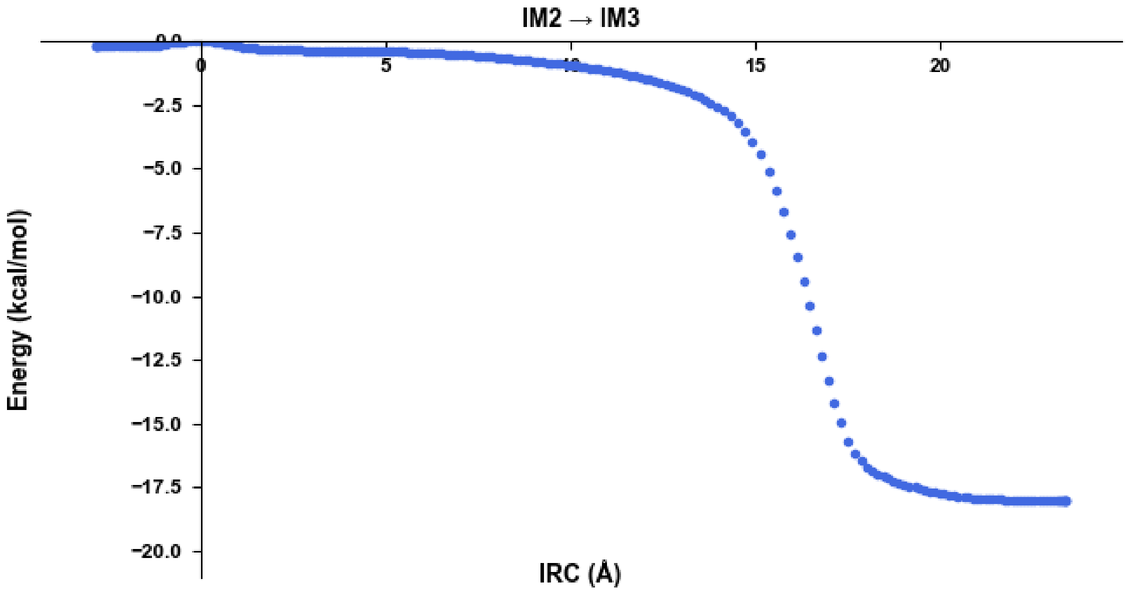

IM2 → IM3
Energy (kcal/mol)
IRC (Å)

2. 3D representation of all computed structures

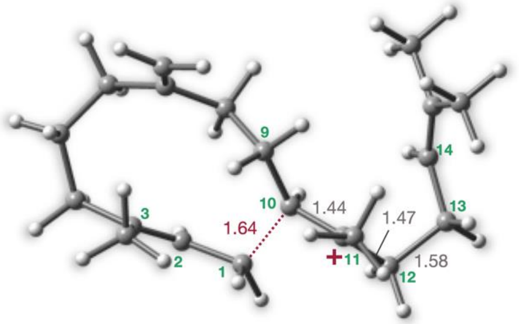

IM1a

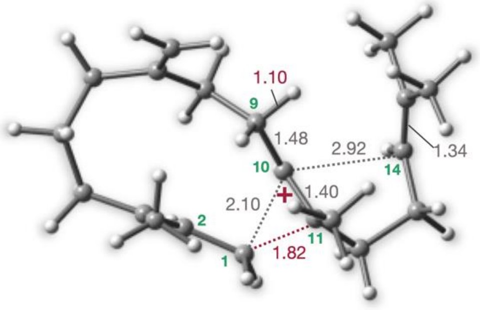

TS_1a-2a

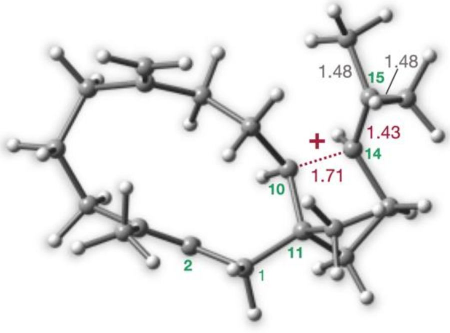

IM2a

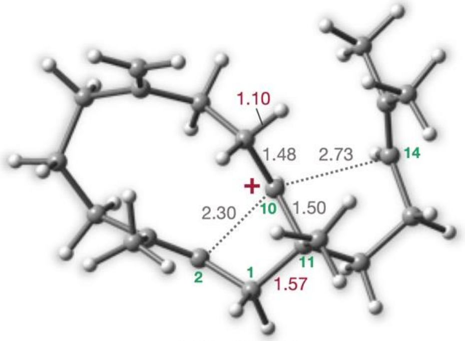

TS_2a-3a

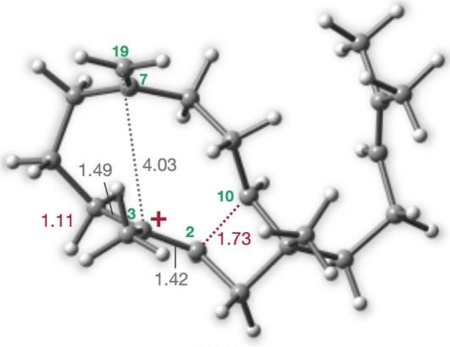

IM3a

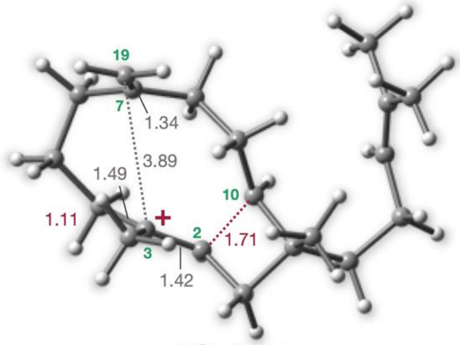

TS_3a-4a

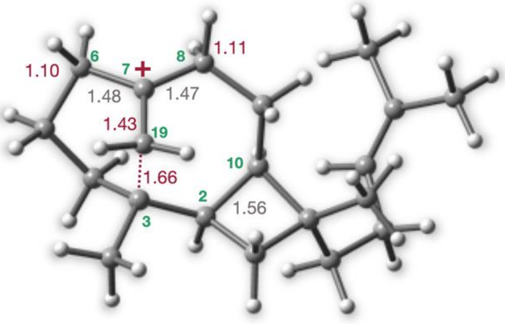

IM4a

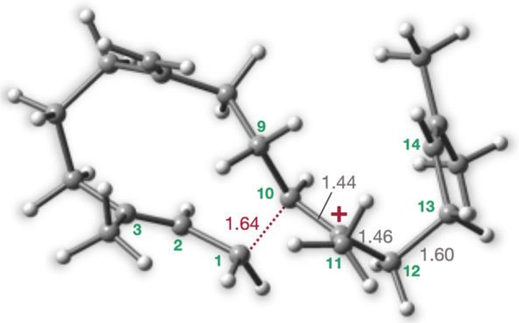

IM1b

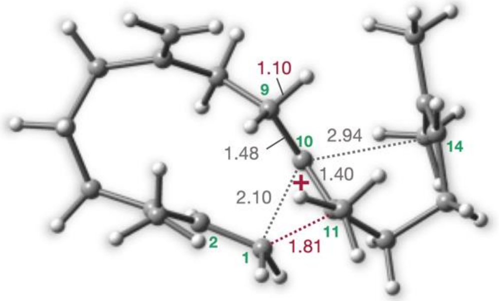

TS_1b-2b

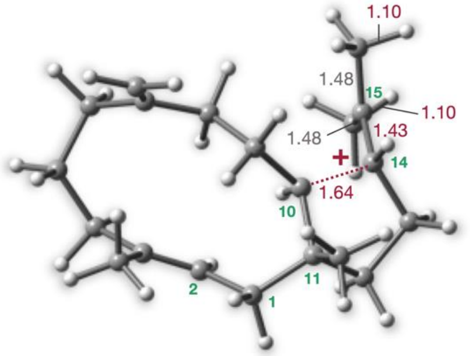

IM2b

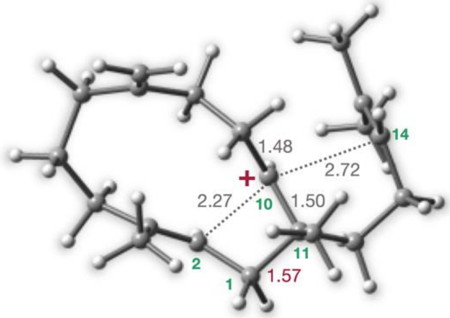

TS_2b-3b

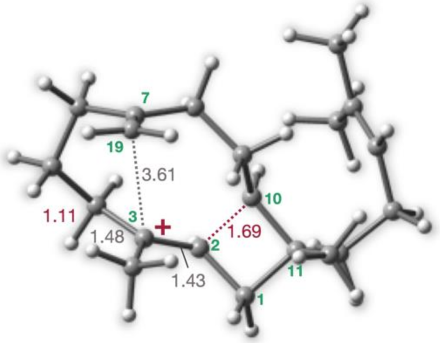

IM3b

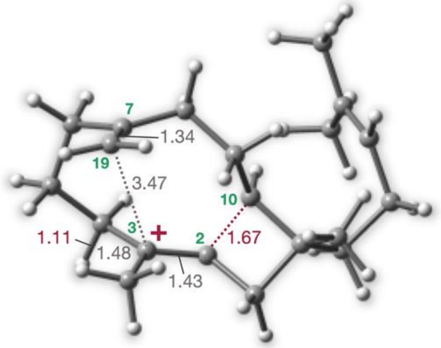

TS_3b-4b

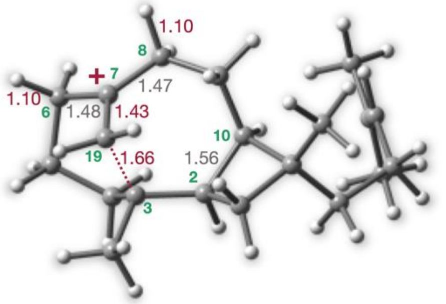

IM4b

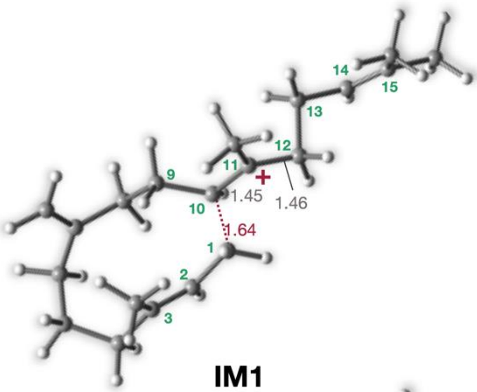

IM1

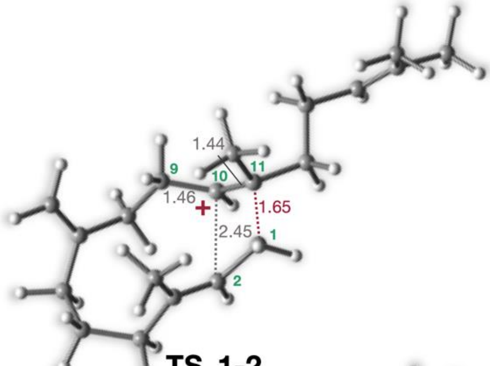

TS_1-2

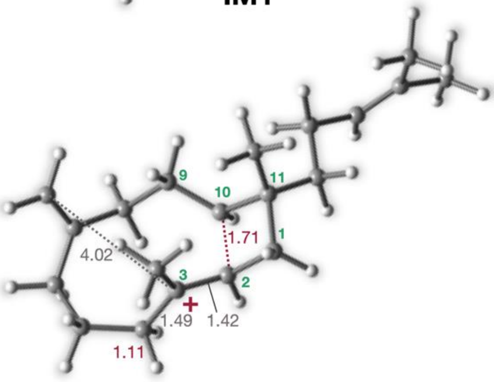

IM2

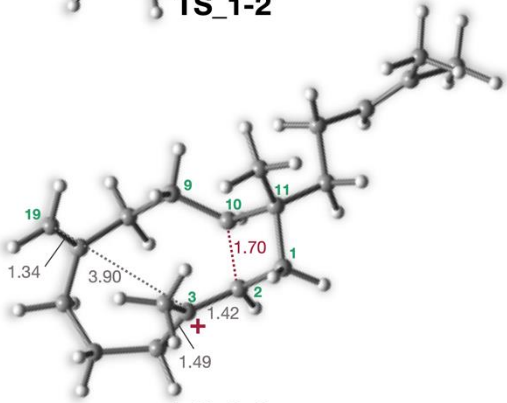

TS_2-3

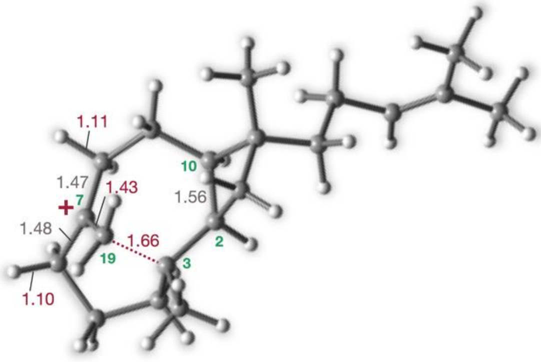

IM3

3. Cartesian coordinates, energies, and imaginary frequencies.

IM1a

Sum of electronic and thermal Free Energies = -781.126136

Number of imaginary frequensies = 0

C	-0.51201500	-2.09154800	-0.33147100
C	-1.82281500	-1.58884400	-0.84363300
C	-2.90480000	-1.27516700	-0.11093200
C	-4.11960100	-0.69686500	-0.79645600
C	-4.47187200	0.73736300	-0.36018500
C	-3.41706400	1.80853100	-0.69917200
C	-2.17602700	1.78114000	0.17794500
C	-0.87678000	1.29546800	-0.44074100
C	-0.04536900	0.36292500	0.46902200
C	0.53016500	-0.82828900	-0.30839100
C	1.67740900	-1.49497700	0.25158600
C	2.66540100	-2.14179500	-0.62343400
C	3.90683800	-1.16790700	-0.62435800
C	3.51345500	0.23316200	-1.00432400
C	3.61652500	1.34422600	-0.25903000
C	3.14576800	2.67054400	-0.79567800
C	4.21234000	1.41641000	1.12314700
C	1.90515900	-1.49320900	1.70584400
C	-2.22682000	2.19761900	1.44602300
C	-3.02870000	-1.45893200	1.37742000
H	-3.89377100	2.78856500	-0.59571100
H	-4.98272100	-1.33987500	-0.58238800
H	4.38993000	-1.21263600	0.35383300
H	0.70609600	-0.58903800	-1.36297100
H	-1.86206100	-1.42163300	-1.91921400
H	-0.59750500	-2.49697800	0.67907700
H	-0.10320900	-2.86463400	-0.98743900
H	0.78061000	0.92346500	0.92277500
H	-0.67344900	-0.00313000	1.28871600
H	-0.26438200	2.16322400	-0.71757900
H	-1.10667300	0.78570900	-1.37923600
H	-1.35371500	2.19170800	2.09413800
H	-3.14840200	2.57769300	1.87908300
H	-3.13358100	1.71188500	-1.75467400
H	-3.97111400	-0.71210400	-1.88218800
H	-4.68136900	0.76647800	0.71568400
H	-5.40682700	1.00786600	-0.86153000
H	-3.96255400	-1.98239100	1.60732400
H	-2.20938300	-2.02964000	1.81878800
H	-3.07327200	-0.48590000	1.87859200
H	2.28759200	-2.24366800	-1.64402200
H	2.98275000	-3.11327500	-0.23334400
H	2.54999700	-2.30551900	2.04295200
H	2.42185800	-0.53739600	1.90809900
H	0.97322800	-1.45215400	2.27486800
H	4.60703400	-1.59015700	-1.35184200
H	3.09154200	0.33493600	-2.00536400
H	3.97962200	3.37826800	-0.85276400
H	2.70543400	2.57714100	-1.79067800
H	2.40246500	3.11547600	-0.12334100
H	3.48289300	1.82507500	1.83347700
H	4.57752100	0.46148200	1.50547700
H	5.05823100	2.11203100	1.12231500

IM2a

Sum of electronic and thermal Free Energies = -781.125171

Number of imaginary frequensies = 0

C	0.04282600	-2.37072100	0.25686100
C	-1.10462500	-1.85380300	-0.57468900
C	-2.30464000	-1.46152000	-0.11552800
C	-3.35917700	-0.92341100	-1.06317900
C	-4.12991800	0.32450500	-0.59243000
C	-3.39741900	1.68029200	-0.64960900
C	-2.18178500	1.78704000	0.24693900
C	-0.84847000	1.51481700	-0.41769100
C	0.18557900	0.79029800	0.46177300
C	1.09424200	-0.13443500	-0.36025100
C	1.37404700	-1.53673300	0.21541500
C	2.39547400	-2.09939700	-0.78081000
C	3.41485000	-0.96989900	-0.98851100
C	2.60376000	0.33534600	-1.00337400
C	2.92374600	1.46495700	-0.19343000
C	2.45031000	2.79886200	-0.61285100
C	3.65024400	1.40576600	1.09083200
C	1.96041500	-1.52134800	1.63191300
C	-2.28549400	2.10418400	1.53972500
C	-2.74509700	-1.58993600	1.32261200
H	-4.11816200	2.45433300	-0.36739200
H	-4.10237900	-1.72024400	-1.20437400
H	4.15069800	-0.96842300	-0.18139700
H	0.59737300	-0.28424400	-1.31969300
H	-0.93521000	-1.78677300	-1.65165800
H	-0.27251100	-2.43469700	1.29999900
H	0.30200100	-3.39389700	-0.04592000
H	0.72517500	1.49040400	1.11084400
H	-0.36568700	0.13737800	1.14790100
H	-0.45162000	2.46612600	-0.79979400
H	-1.04079600	0.89579100	-1.30061900
H	-1.42140000	2.16283100	2.19676400
H	-3.25139200	2.31828700	1.98909700
H	-3.10657100	1.88641000	-1.68796700
H	-2.91629700	-0.73839300	-2.05032500
H	-4.49746700	0.16839300	0.42837600
H	-5.02067600	0.41598600	-1.22273300
H	-3.77748900	-1.95395400	1.35914900

H	-2.13579700	-2.28550700	1.89958100
H	-2.73471300	-0.61753900	1.82787800
H	1.89411700	-2.33582100	-1.72797100
H	2.86656600	-3.01830700	-0.42061100
H	2.00563800	-2.54651900	2.01172900
H	2.97695700	-1.12336300	1.67322900
H	1.33310100	-0.94588500	2.32135400
H	3.96866500	-1.06822500	-1.92460200
H	2.35595800	0.66802400	-2.01334600
H	3.34605400	3.32232100	-0.98699100
H	1.71746300	2.76350200	-1.41836700
H	2.08678200	3.38945400	0.23479300
H	2.88923100	1.29087700	1.87997500
H	4.34219500	0.56915600	1.17148400
H	4.15698100	2.35333100	1.28930900

IM3a

Sum of electronic and thermal Free Energies = -781.124664

Number of imaginary frequensies = 0

C	0.08397200	-2.76754600	0.04265900
C	-0.88102000	-1.82203400	-0.69287400
C	-2.11318900	-1.36411100	-0.16414100
C	-3.14606800	-0.84458200	-1.10078000
C	-4.15244100	0.20950700	-0.61699400
C	-3.62094600	1.65317200	-0.59759000
C	-2.40254200	1.84944500	0.27621200
C	-1.04496000	1.58371000	-0.35824600
C	-0.09771100	0.64639800	0.42668600
C	0.28054800	-0.59019500	-0.35874800
C	1.18065200	-1.69572700	0.25683800
C	2.41879600	-2.00615400	-0.60050600
C	3.57335000	-0.99837300	-0.51149100
C	3.18251000	0.37660500	-0.97647200
C	3.23313900	1.53015800	-0.29424100
C	2.78050900	2.82178900	-0.92549500
C	3.72924900	1.67901600	1.12020900
C	1.54340200	-1.47552700	1.72190500
C	-2.51320400	2.27003600	1.53917300
C	-2.43247200	-1.44121600	1.27608200
H	-4.43203300	2.29820000	-0.24805500
H	-3.70151500	-1.77584100	-1.32983700
H	3.96897000	-0.97907900	0.50724300
H	0.58720400	-0.29737800	-1.36608900
H	-0.90743500	-1.93373600	-1.77733400
H	-0.31904000	-3.18509300	0.96766400
H	0.38980200	-3.59410900	-0.60309500
H	0.85828200	1.14478600	0.62330500
H	-0.51141700	0.39963000	1.40966600
H	-0.55165500	2.55123400	-0.50377000
H	-1.18892400	1.18925400	-1.37136500
H	-1.64283100	2.41992500	2.17380000
H	-3.48092600	2.50449700	1.97408600
H	-3.39080200	1.96350200	-1.62467200
H	-2.68120900	-0.55962500	-2.05144900
H	-4.52964000	-0.05702200	0.37603200
H	-5.01279100	0.16886000	-1.29099700
H	-3.36023500	-2.02024300	1.38814500
H	-1.64714600	-1.89641900	1.87541900
H	-2.66069300	-0.43728000	1.65428400
H	2.10231800	-2.10537400	-1.64960600
H	2.78326600	-2.99616400	-0.29684700
H	2.13015900	-2.32517600	2.08601800
H	2.13894300	-0.56803100	1.85697300
H	0.65582500	-1.39009400	2.35692900
H	4.38581200	-1.37387500	-1.14542600
H	2.83030500	0.42605700	-2.01005900
H	3.61270300	3.53079600	-0.99590800
H	2.37631000	2.66810100	-1.92928500
H	2.01334900	3.30759900	-0.30811000
H	2.92506600	2.04170100	1.77469100
H	4.13039100	0.75840200	1.54523900
H	4.52014500	2.43577600	1.15929600

IM4a

Sum of electronic and thermal Free Energies = -781.149509

Number of imaginary frequensies = 0

C	-0.35878200	-2.22030600	0.43206500
C	-0.97933100	-1.08497300	-0.40308100
C	-2.45662200	-0.70503600	-0.28414900
C	-2.89395700	0.21504300	-1.44934000
C	-3.99052300	1.19603100	-1.03147300
C	-3.41972600	2.13438500	0.08115900
C	-2.40990500	1.34680200	0.81835200
C	-1.02783200	1.83473400	0.94961500
C	0.03365300	0.76480400	1.20047100
C	0.21692200	-0.16988800	0.00682200
C	1.00891200	-1.48339600	0.35601500
C	1.85231700	-1.97967200	-0.83048800
C	3.23102300	-1.30958500	-0.98128800
C	3.15121700	0.17889700	-1.17736000
C	3.68814100	1.15341200	-0.42941100
C	3.48012100	2.60324900	-0.78914400
C	4.55178100	0.94038200	0.78684200
C	1.82954300	-1.50427400	1.64184000
C	-2.77764200	0.01432400	1.17694900
C	-3.32116200	-1.97251800	-0.24919100
H	-4.22645600	2.41236800	0.77301600
H	-3.23605600	-0.41542800	-2.27553800
H	3.85785300	-1.56225700	-0.12232500
H	0.63801200	0.41399100	-0.81743300

H	-0.84587700	-1.33774700	-1.46284900
H	-0.73316600	-2.23823100	1.46425500
H	-0.41499600	-3.23414800	0.02470700
H	0.99073000	1.26488200	1.38488800
H	-0.20309300	0.21970600	2.11946100
H	-1.09416300	2.54162100	1.80157300
H	-0.78678000	2.47131100	0.08763900
H	-2.15824100	-0.48576800	1.91863600
H	-3.84390700	-0.11400900	1.36405200
H	-2.98530000	3.04513600	-0.33528400
H	-2.02893700	0.78163200	-1.82191800
H	-4.86561500	0.65789700	-0.65553700
H	-4.32603500	1.81083600	-1.86834900
H	-3.07161300	-2.58400300	-1.12270300
H	-3.12859500	-2.56693900	0.64740900
H	-4.38786900	-1.73319700	-0.29090800
H	1.28898000	-1.85195800	-1.76539100
H	2.00516400	-3.05971100	-0.70912000
H	2.32893600	-2.47393500	1.74502300
H	2.60131100	-0.72909100	1.64106500
H	1.20943700	-1.36601300	2.53185900
H	3.71951600	-1.75778500	-1.85527900
H	2.58284300	0.48909800	-2.05787700
H	2.83811100	2.71720900	-1.66660500
H	3.03028000	3.15160700	0.04857800
H	4.43686400	3.09314300	-1.00164400
H	4.74984700	-0.10982700	1.00422500
H	5.51747500	1.43879500	0.64778100
H	4.09246000	1.39631200	1.67335600

IM1b

Sum of electronic and thermal Free Energies = -781.128667

Number of imaginary frequensies = 0

C	-0.29611200	-1.60379500	-1.08053400
C	-1.44532700	-0.76898500	-1.54248000
C	-2.72041800	-0.85299600	-1.12623700
C	-3.73538500	0.13604200	-1.64768300
C	-4.33287100	1.05926000	-0.56969600
C	-3.32873700	2.00627100	0.11519500
C	-2.40299600	1.33647100	1.11775400
C	-0.93720200	1.18998500	0.74811600
C	-0.32058200	-0.17947300	1.11401500
C	0.57235500	-0.72282100	-0.00996900
C	1.57529900	-1.69833700	0.34345300
C	2.80739400	-1.78572000	-0.43446600
C	3.89141300	-0.94603300	0.38098400
C	3.51055700	0.48882000	0.59682400
C	3.75168400	1.50398200	-0.24716100
C	3.32941100	2.90764600	0.09126700
C	4.45165800	1.35682500	-1.57094300
C	1.39496200	-2.57044800	1.51903400
C	-2.86502900	0.91625300	2.29836600
C	-3.25304700	-1.87677500	-0.16064300
H	-3.90240200	2.77998900	0.63556100
H	-4.55811700	-0.41865600	-2.11627700
H	4.07012200	-1.45121800	1.33429900
H	1.02946900	0.07867100	-0.60389600
H	-1.18747200	0.01242400	-2.25631400
H	-0.63003400	-2.52105800	-0.59010500
H	0.35767600	-1.87023000	-1.91501500
H	0.26572800	-0.08854100	2.03511900
H	-1.12316200	-0.89579600	1.32181100
H	-0.36206800	1.98430700	1.24091300
H	-0.82752900	1.36845200	-0.32413400
H	-2.22431300	0.43904800	3.03594200
H	-3.90672900	1.05144800	2.57758300
H	-2.74001600	2.52200000	-0.65376200
H	-3.27890700	0.75275400	-2.43026100
H	-4.84821200	0.46648700	0.19529800
H	-5.10234000	1.66847600	-1.05492800
H	-4.15243600	-2.34264500	-0.57661200
H	-2.54256600	-2.67220400	0.07204000
H	-3.54455500	-1.39639300	0.77977000
H	2.70333900	-1.31688500	-1.41638100
H	3.17242400	-2.81207200	-0.52089200
H	1.99804300	-3.47775000	1.47691800
H	1.72620500	-1.97964400	2.39068300
H	0.34155600	-2.79724100	1.70437100
H	4.80581300	-1.04365400	-0.20824400
H	2.98969500	0.71557800	1.52838900
H	2.65282500	3.30214100	-0.67511300
H	2.83062200	2.96283500	1.06121100
H	4.20058000	3.57105100	0.11364400
H	5.35850400	1.97073800	-1.58530300
H	4.73536100	0.33043100	-1.81000800
H	3.81073300	1.73012600	-2.37748500

IM2b

Sum of electronic and thermal Free Energies = -781.136011

Number of imaginary frequensies = 0

C	0.15797100	-2.43863600	-0.12582500
C	-0.85375000	-1.68606300	-0.95239100
C	-2.12546500	-1.42273700	-0.60906900
C	-3.02601900	-0.61810800	-1.52692000
C	-3.91440900	0.44909200	-0.85905000
C	-3.24147200	1.75841600	-0.39930800
C	-2.20011900	1.59482400	0.68709300
C	-0.76766100	1.47596500	0.20991600
C	0.12363200	0.51918000	1.01966300
C	1.20302600	-0.11425700	0.14719000

C	1.44083400	-1.64475600	0.28978300
C	2.62418500	-1.85078500	-0.66308100
C	3.58932600	-0.68869300	-0.36042600
C	2.74525000	0.42839700	0.30527400
C	2.59793200	1.69977000	-0.33041600
C	2.42276100	2.91327400	0.49198400
C	2.59356900	1.85369500	-1.79884900
C	1.82458500	-2.04150700	1.71893500
C	-2.52747200	1.55520600	1.98058100
C	-2.79318400	-1.94943100	0.63778700
H	-4.03214800	2.42409300	-0.03891700
H	-3.70351200	-1.33071100	-2.01728700
H	4.36017300	-0.98416000	0.35522300
H	0.93615200	0.03605200	-0.90306200
H	-0.50800300	-1.30983300	-1.91827800
H	-0.31826400	-2.79842400	0.78823000
H	0.49928200	-3.33315500	-0.66396100
H	0.55012700	1.00206000	1.90816700
H	-0.50748700	-0.29048400	1.40318500
H	-0.32973800	2.48575200	0.16929900
H	-0.79826500	1.11932400	-0.82570300
H	-1.78744900	1.41449100	2.76419000
H	-3.56013900	1.66320400	2.30089100
H	-2.78892200	2.25208000	-1.26978400
H	-2.43108200	-0.16074000	-2.32829200
H	-4.44121700	0.00866600	-0.00475200
H	-4.69004600	0.72585200	-1.58087100
H	-3.80122800	-2.30212000	0.39441200
H	-2.25894400	-2.78285900	1.09356500
H	-2.90722100	-1.15869000	1.38808500
H	2.27194600	-1.80078400	-1.70121300
H	3.10010900	-2.82580500	-0.52706200
H	1.90874300	-3.13080000	1.78010600
H	2.78511900	-1.62519500	2.03879700
H	1.05976600	-1.72986400	2.43736900
H	4.11497200	-0.33616200	-1.25086100
H	2.92447300	0.53423300	1.37814200
H	1.96771600	3.74544600	-0.04557000
H	1.89311000	2.69994200	1.42492000
H	3.44283600	3.21019800	0.78934600
H	3.43557100	2.51039800	-2.06436500
H	2.68069900	0.91483200	-2.34369200
H	1.69174200	2.39608800	-2.10748600

IM3b

Sum of electronic and thermal Free Energies = -781.122496

Number of imaginary frequensies = 0

C	0.18879500	-2.54303600	-0.48054000
C	-0.57749100	-1.32714200	-1.02202700
C	-1.97064000	-1.10283300	-0.80587000
C	-2.67854700	-0.15885800	-1.70503300
C	-3.86306900	0.66448100	-1.18507200
C	-3.44025100	1.83154600	-0.28043000
C	-2.57800000	1.37642400	0.87793900
C	-1.07376100	1.56374200	0.74823000
C	-0.20658500	0.36323200	1.17265800
C	0.38831300	-0.38789800	0.00274300
C	1.23659300	-1.67131100	0.25382000
C	2.56511400	-1.63218900	-0.51763100
C	3.69261900	-0.81510400	0.13973200
C	3.29304800	0.57835200	0.56809000
C	3.12239600	1.63961500	-0.23268000
C	2.68525200	2.97326700	0.31240800
C	3.32620600	1.59814100	-1.72425000
C	1.43772200	-2.06691100	1.71317400
C	-3.13649000	0.87106600	1.98209300
C	-2.70796100	-1.92311500	0.17941400
H	-4.34718700	2.31533400	0.09340800
H	-3.05805100	-0.86205500	-2.47497200
H	4.06144500	-1.36077200	1.01374100
H	0.87307400	0.32766000	-0.66417100
H	-0.27911100	-1.01839100	-2.02634700
H	-0.39972300	-3.15854800	0.20393900
H	0.58583100	-3.18193900	-1.27402200
H	0.66605100	0.71264500	1.73765500
H	-0.75616500	-0.29701600	1.85158900
H	-0.79982700	2.43999200	1.34704600
H	-0.82607700	1.83648900	-0.28569300
H	-2.54791000	0.54145700	2.83432800
H	-4.21679500	0.80198000	2.08398000
H	-2.90532300	2.57980700	-0.87614600
H	-1.94982600	0.47105200	-2.22851500
H	-4.56812200	0.02058900	-0.65084500
H	-4.40027500	1.05412400	-2.05434000
H	-2.74592500	-2.94465600	-0.23211200
H	-2.14958700	-2.00112600	1.11746700
H	-3.71554300	-1.57017900	0.37987300
H	2.36970700	-1.24447900	-1.52838800
H	2.90950500	-2.66658000	-0.64614600
H	1.97345000	-3.02109500	1.75798800
H	2.02435200	-1.32685300	2.26406200
H	0.48516200	-2.19956900	2.23541400
H	4.53342400	-0.77507000	-0.56110700
H	3.13141400	0.72811800	1.63645800
H	2.53822000	2.94257700	1.39510200
H	3.42768000	3.74745600	0.09000500
H	1.74762400	3.29637200	-0.15891500
H	3.74286200	0.65136300	-2.07280300
H	2.37638100	1.77009700	-2.24930900
H	3.99968500	2.40239800	-2.03881900

IM4b

Sum of electronic and thermal Free Energies = -781.15014
Number of imaginary frequensies = 0

C	-0.34360900	-2.27670500	-0.34416000
C	-0.77059500	-0.83620900	-0.68219700
C	-2.24190000	-0.43255700	-0.79644200
C	-2.39147000	0.95926500	-1.45719700
C	-3.61041900	1.71995400	-0.93308400
C	-3.41594900	1.97486700	0.59684900
C	-2.61080000	0.84787300	1.11198500
C	-1.34575100	1.09230200	1.82270900
C	-0.33918300	-0.05743300	1.80320600
C	0.22184500	-0.31999500	0.40667300
C	0.96088400	-1.70432400	0.27941000
C	2.09613900	-1.65658000	-0.75817500
C	3.44317400	-1.11258000	-0.24691300
C	3.36854400	0.26478400	0.36040300
C	3.59355600	1.43333100	-0.25589100
C	3.48748700	2.73955600	0.48756900
C	3.96996200	1.56883800	-1.70780000
C	1.42921700	-2.39577200	1.55620400
C	-2.99467300	-0.45866300	0.68318300
C	-3.01125700	-1.49152400	-1.59795200
H	-4.39494300	1.96769400	1.09516600
H	-2.46109700	0.81995400	-2.54017500
H	3.85170000	-1.80644500	0.49599000
H	0.82020700	0.54529100	0.10178300
H	-0.34150600	-0.58031400	-1.65924100
H	-0.97634000	-2.73423300	0.42797500
H	-0.24337100	-2.98219700	-1.17437100
H	0.49185200	0.20564200	2.46556800
H	-0.79411100	-0.95247400	2.23894800
H	-1.68129100	1.31334500	2.85636900
H	-0.91013900	2.03677800	1.47034100
H	-2.58069400	-1.30064600	1.23391100
H	-4.06246800	-0.56549400	0.49192800
H	-2.92936200	2.93206400	0.79336800
H	-1.48833400	1.55813600	-1.27342900
H	-4.52553200	1.14542300	-1.10405600
H	-3.73573600	2.68125900	-1.43419100
H	-2.49742000	-1.64087900	-2.55338800
H	-3.04018400	-2.45041300	-1.07449500
H	-4.03676300	-1.17221700	-1.80573400
H	1.78293600	-1.05453000	-1.62197400
H	2.25392300	-2.67787600	-1.12897100
H	1.89484900	-3.35464100	1.30383900
H	2.17184800	-1.80216100	2.09834400
H	0.60294600	-2.60934600	2.23978200
H	4.14961600	-1.12492600	-1.08158200
H	3.10177200	0.30619500	1.41904100
H	3.21817600	2.58988100	1.53652600
H	4.43903200	3.28196600	0.45503000
H	2.73985200	3.39396800	0.02221000
H	3.99584100	0.61570900	-2.23764700
H	3.25885900	2.22479200	-2.22378500
H	4.95466400	2.04012400	-1.80336300

IM1

Sum of electronic and thermal Free Energies = -781.13016
Number of imaginary frequensies = 0

C	-0.51930100	-1.48592400	0.07925800
C	-1.84923100	-1.54777300	-0.59731900
C	-3.05540200	-1.52620500	-0.00493700
C	-4.29915800	-1.51769100	-0.86101800
C	-5.14955700	-0.24059200	-0.72970500
C	-4.46290700	1.05701500	-1.19756200
C	-3.40062600	1.59178700	-0.25094000
C	-1.94777000	1.50066900	-0.68471900
C	-0.97450400	1.06644200	0.43527900
C	0.05674500	0.04121300	-0.05691900
C	1.27421100	-0.11092200	0.70675000
C	2.50089900	-0.55626000	0.05397800
C	3.35542100	0.76527200	-0.19387500
C	4.58296900	0.42005000	-0.98585500
C	5.76660000	0.05777600	-0.46920300
C	6.93840900	-0.24210700	-1.36291800
C	6.04561600	-0.07747900	1.00313500
C	1.30322500	0.22242000	2.14377500
C	-3.74280300	2.15099700	0.91269000
C	-3.29307900	-1.51473800	1.48082900
H	-5.23860500	1.82038900	-1.31598000
H	-4.92441900	-2.37488300	-0.58098900
H	2.74061500	1.48388700	-0.74465700
H	0.28694400	0.15689100	-1.12342500
H	-1.81058400	-1.56764500	-1.68571700
H	-0.59075900	-1.73865200	1.13974400
H	0.19591400	-2.16267800	-0.39497100
H	-0.45503900	1.94321000	0.83717300
H	-1.54654600	0.63374300	1.26358000
H	-1.63163100	2.47515800	-1.07727700
H	-1.87719800	0.80587300	-1.52478300
H	-3.00482000	2.54791900	1.60556600
H	-4.78449300	2.24893100	1.20707400
H	-4.03235300	0.89630900	-2.19384100
H	-4.02229900	-1.65360600	-1.91266400
H	-5.48968200	-0.11547500	0.30514800
H	-6.05125900	-0.38810100	-1.33286800
H	-4.02156500	-2.28880100	1.74350300
H	-2.39164100	-1.69108600	2.07086800
H	-3.71836800	-0.55373000	1.78999100
H	2.31149600	-1.01903100	-0.91753300
H	3.10145500	-1.21433500	0.68717600
H	2.12467100	-0.25807800	2.67635200

H	1.44556100	1.31498200	2.20515500
H	0.34337000	0.02641700	2.62952500
H	3.60360800	1.20937700	0.77310100
H	4.47506000	0.46518600	-2.06760400
H	7.29614200	-1.26385300	-1.19551400
H	6.68619500	-0.13006200	-2.41891000
H	7.77297000	0.42875200	-1.13250000
H	6.84873600	0.60582000	1.29929000
H	5.18016000	0.12398500	1.63826700
H	6.39982300	-1.09050200	1.22338200

IM2

Sum of electronic and thermal Free Energies = -781.127151

Number of imaginary frequensies = 0

C	0.09076500	-2.17393100	-0.06917500
C	-1.02912300	-1.41127300	-0.80582100
C	-2.37524200	-1.34012900	-0.35546300
C	-3.44011300	-1.01857100	-1.34029300
C	-4.70926300	-0.28573200	-0.88127500
C	-4.55517900	1.23908400	-0.75151000
C	-3.48500300	1.66410700	0.22862700
C	-2.06602000	1.80287800	-0.30624900
C	-0.96586800	1.09032900	0.51291100
C	-0.24664100	0.00864100	-0.26172300
C	0.81125300	-0.89499000	0.42250900
C	2.19501500	-0.71625700	-0.21273300
C	2.84728400	0.65004400	0.04073000
C	4.12558100	0.80749800	-0.74213200
C	5.35867300	0.50754900	-0.31380800
C	6.55946200	0.69617000	-1.20227900
C	5.67348800	-0.03200900	1.05676300
C	0.86829900	-0.78486700	1.94428200
C	-3.78169100	1.94131900	1.50124300
C	-2.76003100	-1.62639600	1.04013900
H	-5.52199400	1.64279800	-0.43803400
H	-3.72480600	-2.04054800	-1.66348800
H	2.15045200	1.44881700	-0.25181500
H	0.11462600	0.42438900	-1.20791000
H	-0.96297200	-1.41774600	-1.89423100
H	-0.26229600	-2.83208800	0.72801900
H	0.67831000	-2.76830200	-0.77248000
H	-0.17491600	1.79788600	0.79339500
H	-1.36467800	0.71373200	1.46047700
H	-1.83955800	2.87307500	-0.36287300
H	-2.03053600	1.45250800	-1.34494500
H	-3.02362500	2.25552200	2.21487300
H	-4.80388600	1.89106100	1.86619800
H	-4.34123000	1.66385400	-1.74032000
H	-3.00300700	-0.55762100	-2.23299000
H	-5.06456500	-0.70091600	0.06776800
H	-5.48932400	-0.49363800	-1.61892300
H	-3.58210400	-2.35426200	1.03734700
H	-1.94164100	-1.99571900	1.65353500
H	-3.17978100	-0.71285100	1.48328900
H	2.11169600	-0.87468200	-1.29775700
H	2.85802300	-1.50865200	0.15998500
H	1.59232100	-1.50962000	2.33022600
H	1.18073700	0.20998900	2.27229400
H	-0.09589400	-1.00074300	2.41495100
H	3.03334800	0.78192800	1.11070300
H	4.01526200	1.17430000	-1.76238800
H	7.08548400	-0.25381400	-1.34862200
H	6.28267300	1.09303700	-2.18132800
H	7.27371700	1.38637300	-0.73945700
H	6.33705800	0.65544300	1.59319000
H	4.78830100	-0.20137000	1.67313200
H	6.21093200	-0.98293700	0.96972500

IM3

Sum of electronic and thermal Free Energies = -781.151186

Number of imaginary frequensies = 0

C	0.20991200	0.37391700	-1.73701800
C	0.95236100	-0.40753100	-0.63543400
C	2.43190000	-0.77650600	-0.76094400
C	2.84205600	-1.81720500	0.30887100
C	4.29945800	-1.65601400	0.74377400
C	4.46721300	-0.24925900	1.40494200
C	3.48663800	0.64190500	0.75106300
C	2.49172700	1.37665700	1.54851900
C	1.21372100	1.77999700	0.81304700
C	0.35504000	0.57825400	0.41923700
C	-0.74988000	0.92077600	-0.64401800
C	-1.97960300	0.01570500	-0.47887600
C	-2.89494900	0.38741000	0.69749900
C	-3.95295000	-0.65966900	0.93021200
C	-5.19107700	-0.66806100	0.41947900
C	-6.15171500	-1.78827400	0.71959100
C	-5.74493700	0.40520600	-0.48069400
C	-1.17339900	2.37834600	-0.79522800
C	3.40490000	0.56590900	-0.67196300
C	2.71284000	-1.31686700	-2.16998900
H	5.47879200	0.12941500	1.20462200
H	2.67098400	-2.81720500	-0.10077900
H	-2.29268600	0.49859300	1.60990000
H	-0.01700200	0.09742600	1.33343200
H	0.44359100	-1.37057400	-0.50640400
H	0.81839900	1.18150700	-2.16534200
H	-0.23054800	-0.20239900	-2.55582700
H	0.62919800	2.42642500	1.47448300
H	1.46755700	2.39680100	-0.05473300

H	3.05566400	2.27020400	1.88516400
H	2.28722000	0.82031700	2.47268800
H	2.88015500	1.37156500	-1.18096100
H	4.34624800	0.30579200	-1.15605300
H	4.31309800	-0.28410600	2.48503700
H	2.19227200	-1.72208500	1.19022400
H	4.97028000	-1.74885800	-0.11533300
H	4.59449000	-2.41869700	1.46637900
H	2.01218400	-2.13382100	-2.37203700
H	2.56688900	-0.54784500	-2.93260200
H	3.73075000	-1.70859200	-2.25449800
H	-1.66634600	-1.03037900	-0.36106600
H	-2.56695300	0.05093200	-1.40636800
H	-1.94306100	2.45803300	-1.57053800
H	-1.59629800	2.77839800	0.13145400
H	-0.34379100	3.02461800	-1.09564900
H	-3.35487400	1.36292900	0.51565000
H	-3.65630100	-1.50300300	1.55440900
H	-5.70858300	-2.53775000	1.37913300
H	-7.05723300	-1.39941800	1.19903300
H	-6.47020800	-2.28385300	-0.20447600
H	-5.02154100	1.18646700	-0.72127300
H	-6.08939400	-0.03712500	-1.42227800
H	-6.61848600	0.87811200	-0.01779600

TS_1a-2a
Sum of electronic and thermal Free Energies = -781.110369
Number of imaginary frequensies = 1
The magnitude of the imaginary frequency: -198.6041

C	-0.15994700	-2.24649000	-0.42939500
C	-1.45516500	-1.64572600	-0.87879400
C	-2.53154300	-1.37250000	-0.12170800
C	-3.79693200	-0.88091600	-0.78690200
C	-4.39105900	0.43259300	-0.25041100
C	-3.61652700	1.71774200	-0.59938800
C	-2.29385600	1.86594400	0.12415900
C	-1.03127000	1.48212600	-0.62750900
C	-0.03316600	0.70638600	0.25103600
C	0.74620400	-0.34851500	-0.43950800
C	1.35425500	-1.44247700	0.18285100
C	2.52679400	-2.08241300	-0.53755900
C	3.79659700	-1.22317200	-0.32417900
C	3.58770700	0.16102300	-0.86791800
C	3.50883300	1.31873700	-0.18913400
C	3.23425500	2.61464100	-0.90571900
C	3.70637500	1.46494900	1.29630300
C	1.40329400	-1.52020200	1.69349400
C	-2.24588800	2.32387100	1.37841800
C	-2.59953900	-1.56761500	1.36707500
H	-4.25419600	2.56895200	-0.34194400
H	-4.55260600	-1.66659500	-0.65218000
H	4.07212800	-1.21494300	0.73325300
H	0.88033300	-0.25962900	-1.51975600
H	-1.52130400	-1.46038200	-1.94902800
H	-0.27634800	-2.83693200	0.47966000
H	0.28151700	-2.83535400	-1.23051600
H	0.73279700	1.37547500	0.67737200
H	-0.54277000	0.27445800	1.11764600
H	-0.55430300	2.38742100	-1.02223500
H	-1.30476000	0.87906400	-1.49672400
H	-1.31268500	2.43492700	1.92528100
H	-3.14958700	2.62744900	1.89966400
H	-3.45971200	1.76141100	-1.68447500
H	-3.63277800	-0.78955300	-1.86702200
H	-4.51963800	0.37575600	0.83717400
H	-5.39682700	0.52544000	-0.67245100
H	-3.52996200	-2.07612700	1.63944400
H	-1.76624600	-2.15150400	1.76571000
H	-2.60928600	-0.59630200	1.87618700
H	2.32068400	-2.13828700	-1.61260800
H	2.68162000	-3.10287100	-0.17270900
H	1.69062400	-2.52667000	2.00864000
H	2.15659700	-0.81925700	2.06621000
H	0.45232800	-1.26635600	2.16512700
H	4.61668400	-1.71530400	-0.85744500
H	3.46770000	0.21123700	-1.95239400
H	4.07036200	3.31021100	-0.77675400
H	3.07196800	2.46712700	-1.97573500
H	2.35161000	3.11159700	-0.48199800
H	2.79224800	1.83917700	1.77674400
H	4.00798000	0.54282600	1.79456300
H	4.47929500	2.21562500	1.49201500

TS_2a-3a
Sum of electronic and thermal Free Energies = -781.1152
Number of imaginary frequensies = 1
The magnitude of the imaginary frequency: -291.0430

C	0.11521500	-2.61301400	-0.02735600
C	-1.02808300	-1.89986000	-0.74357700
C	-2.18148800	-1.45217300	-0.18292600
C	-3.29846800	-0.93128600	-1.06031300
C	-4.16534400	0.22344600	-0.52722700
C	-3.56668200	1.64158900	-0.58686000
C	-2.32607200	1.83588200	0.25261200
C	-1.00684100	1.51468000	-0.42322800
C	-0.02306000	0.71264400	0.44953600
C	0.63654700	-0.36877200	-0.30776900
C	1.28224600	-1.59808100	0.24978200
C	2.51744000	-1.99831500	-0.58252500
C	3.65897500	-0.97715500	-0.51482000

C	3.17420200	0.35984600	-0.99170900
C	3.10112100	1.51674700	-0.30111000
C	2.55548800	2.76290700	-0.94164100
C	3.55317300	1.70058700	1.11976700
C	1.59650300	-1.48761200	1.74193400
C	-2.38324300	2.27569400	1.51217500
C	-2.47645000	-1.54573400	1.28366900
H	-4.33943500	2.33991800	-0.25202900
H	-3.96895400	-1.79162500	-1.20478400
H	4.06330200	-0.92718900	0.49886600
H	0.67795100	-0.24803500	-1.38903400
H	-0.96443700	-1.86857100	-1.83157800
H	-0.21324500	-3.04607700	0.91889800
H	0.48293200	-3.42973500	-0.65421800
H	0.77918400	1.34487700	0.86059000
H	-0.51185000	0.29307400	1.33700000
H	-0.53760200	2.44834500	-0.75489700
H	-1.21337300	0.94546300	-1.33559800
H	-1.49226300	2.41020500	2.12136600
H	-3.33125000	2.53420000	1.97504900
H	-3.34563100	1.89382800	-1.63221400
H	-2.90829900	-0.68852000	-2.05627100
H	-4.47735700	0.01189200	0.50170200
H	-5.08316200	0.23478800	-1.12325600
H	-3.44135100	-2.04358200	1.43469500
H	-1.71804500	-2.09096200	1.84585900
H	-2.57735700	-0.54376400	1.71754900
H	2.20549700	-2.12672300	-1.62850700
H	2.85373200	-2.98075700	-0.23315400
H	2.06626900	-2.41539800	2.08092300
H	2.28133700	-0.66152000	1.95279400
H	0.69146000	-1.33862500	2.33795700
H	4.47286800	-1.32569200	-1.16029900
H	2.87399100	0.39254500	-2.04167700
H	3.34012300	3.52347300	-1.02076600
H	2.15558700	2.57622000	-1.94124800
H	1.76606900	3.20484500	-0.31873800
H	2.70447900	1.96836000	1.76563600
H	4.04921200	0.82770400	1.54362000
H	4.24854300	2.54492100	1.17529200

TS_3a-4a

Sum of electronic and thermal Free Energies = -781.123739

Number of imaginary frequensies = 1

The magnitude of the imaginary frequency: -100.3521

C	0.05211500	-2.72719500	0.05828500
C	-0.88132600	-1.76161600	-0.69368800
C	-2.13080800	-1.31459600	-0.18812000
C	-3.13829700	-0.78934900	-1.14692100
C	-4.16333100	0.25966300	-0.69739100
C	-3.60690700	1.68985100	-0.59939000
C	-2.41613400	1.80841500	0.32599400
C	-1.03707200	1.62134700	-0.28983000
C	-0.08251200	0.66981200	0.46563100
C	0.28382000	-0.55514600	-0.34511200
C	1.17406600	-1.67850000	0.25700100
C	2.39004200	-2.00900200	-0.62408600
C	3.56440100	-1.02251500	-0.54842600
C	3.18919000	0.36439000	-0.99051300
C	3.26503400	1.50831300	-0.29456700
C	2.82058800	2.81354500	-0.90336900
C	3.78421100	1.63329100	1.11396500
C	1.57097400	-1.47031400	1.71503800
C	-2.57370500	2.10091100	1.61979600
C	-2.48079400	-1.47790200	1.23916200
H	-4.41523300	2.33815600	-0.24950900
H	-3.68380600	-1.72136300	-1.39852900
H	3.97999900	-1.02118800	0.46251100
H	0.60327900	-0.24072700	-1.34163700
H	-0.88886800	-1.87416300	-1.77860600
H	-0.36568100	-3.11439000	0.99019700
H	0.33552500	-3.57483000	-0.57048500
H	0.87696200	1.16267000	0.65980800
H	-0.49009100	0.40744500	1.44699800
H	-0.57345200	2.61115900	-0.36905300
H	-1.14482400	1.27972900	-1.32676900
H	-1.72931800	2.19272300	2.29849600
H	-3.55875500	2.28664600	2.03989900
H	-3.33331500	2.03914600	-1.60253100
H	-2.64314600	-0.49902600	-2.08069000
H	-4.60755000	-0.03222400	0.25967900
H	-4.97855500	0.24943200	-1.42653600
H	-3.08501500	-2.40000000	1.29334200
H	-1.60893600	-1.62735100	1.87502600
H	-3.09122900	-0.65909400	1.61698200
H	2.05384400	-2.09594600	-1.66820700
H	2.74202000	-3.00715400	-0.33261800
H	2.15328700	-2.32995000	2.06267800
H	2.18205700	-0.57219900	1.84182500
H	0.69914900	-1.37587600	2.37015700
H	4.35743100	-1.40546100	-1.20212900
H	2.82356300	0.43234000	-2.01834500
H	2.40176500	2.67767800	-1.90371800
H	2.06695900	3.30022600	-0.27028300
H	3.66036000	3.51339300	-0.97538900
H	4.18292700	0.70319700	1.52031300
H	4.58322700	2.38159600	1.15049800
H	2.99409900	1.99499600	1.78586100

TS_1b-2b

Sum of electronic and thermal Free Energies = -781.109647

Number of imaginary frequensies = 1
The magnitude of the imaginary frequency: -196.7772

C	-0.01723100	-2.02044300	-0.72142300
C	-1.26331100	-1.34221300	-1.20022500
C	-2.45700000	-1.30212600	-0.58437800
C	-3.63434300	-0.67536200	-1.29556800
C	-4.37506500	0.44381000	-0.54460900
C	-3.62729800	1.78528900	-0.42729900
C	-2.44247600	1.75869500	0.51653000
C	-1.05786200	1.61323100	-0.09094300
C	-0.15590700	0.65887800	0.71403600
C	0.76858300	-0.18427400	-0.07859000
C	1.31646800	-1.40003700	0.34107400
C	2.62568000	-1.81397100	-0.30126700
C	3.80538500	-1.12979600	0.43465100
C	3.56521800	0.34335800	0.66105400
C	3.61371800	1.30743700	-0.27360600
C	3.31207900	2.74237800	0.06636000
C	3.98064200	1.05724900	-1.71239000
C	1.11762400	-1.87410200	1.76387400
C	-2.61596600	1.86989400	1.83664400
C	-2.73636000	-1.88849900	0.77095900
H	-4.34200800	2.53354700	-0.07128100
H	-4.35537800	-1.48138800	-1.48748200
H	3.97381300	-1.62622700	1.39442800
H	1.08310800	0.18182800	-1.05873300
H	-1.17653500	-0.87784300	-2.18049300
H	-0.23696300	-2.84781100	-0.04573600
H	0.58234400	-2.35030500	-1.56735700
H	0.51359600	1.21665400	1.39035700
H	-0.75880500	0.02554400	1.37146800
H	-0.58802100	2.60177700	-0.15670400
H	-1.15579000	1.25305000	-1.11785800
H	-1.78690800	1.85097300	2.53979800
H	-3.60608400	2.00875100	2.26199000
H	-3.30737800	2.10894000	-1.42569700
H	-3.31552700	-0.30327000	-2.27632100
H	-4.66230300	0.10423400	0.45781100
H	-5.30936700	0.62881300	-1.08422900
H	-3.67087600	-2.45820500	0.75187700
H	-1.94347400	-2.55141900	1.12611400
H	-2.86824500	-1.08854800	1.50965100
H	2.63024200	-1.49887000	-1.35064100
H	2.73555500	-2.90336600	-0.27562800
H	1.39749100	-2.92738000	1.85015000
H	1.76754900	-1.29669500	2.42999100
H	0.09104200	-1.75702800	2.11493500
H	4.70903000	-1.29937900	-0.15931900
H	3.31732100	0.64382600	1.67989200
H	2.50020600	3.12878600	-0.56233500
H	3.03258900	2.86456800	1.11556600
H	4.18451900	3.37464300	-0.13020800
H	4.78217700	1.73779000	-2.01769500
H	4.31674900	0.03616000	-1.90160100
H	3.13087400	1.27148100	-2.37401700

TS_2b-3b
Sum of electronic and thermal Free Energies = -781.114363
Number of imaginary frequensies = 1
The magnitude of the imaginary frequency: -305.2444

C	0.33989200	-2.46460200	-0.51794500
C	-0.74243100	-1.58823300	-1.14306800
C	-1.99732500	-1.37580300	-0.66322000
C	-3.01897900	-0.66161600	-1.51860100
C	-4.05115400	0.24402700	-0.82525300
C	-3.57078500	1.64100900	-0.39230600
C	-2.47844500	1.62678100	0.65052600
C	-1.05268800	1.58150600	0.13237200
C	-0.13580100	0.60149900	0.88940100
C	0.68781400	-0.23082900	-0.01272500
C	1.31299200	-1.55827300	0.30912200
C	2.73550900	-1.64550100	-0.27618300
C	3.74099700	-0.72407400	0.42983300
C	3.17811800	0.66138200	0.62269200
C	3.00237500	1.58955100	-0.34113500
C	2.42664100	2.94291000	-0.02879200
C	3.39151800	1.37966300	-1.77909100
C	1.28345400	-1.90158700	1.79860000
C	-2.75008400	1.66846100	1.95738500
C	-2.48277800	-1.92596300	0.64251000
H	-4.43626200	2.18197400	0.00156500
H	-3.58143400	-1.46992400	-2.01007100
H	4.00726700	-1.14420700	1.40346300
H	0.91350000	0.18100700	-0.99493600
H	-0.53693100	-1.22294000	-2.14930400
H	-0.08493200	-3.23351000	0.13076900
H	0.89155000	-2.97218100	-1.31350000
H	0.57086200	1.13166900	1.54456000
H	-0.70481400	-0.04494300	1.56729500
H	-0.63268900	2.59278000	0.17435700
H	-1.06983200	1.30770200	-0.92822800
H	-1.96956800	1.65387200	2.71459900
H	-3.77291100	1.73825600	2.31628600
H	-3.22926900	2.19390100	-1.27707200
H	-2.51464900	-0.11786700	-2.32667800
H	-4.47791400	-0.27074900	0.04303600
H	-4.87750900	0.38592300	-1.52858000
H	-3.43147200	-2.45373400	0.49205400
H	-1.77694700	-2.61288200	1.11009900
H	-2.69416200	-1.10963900	1.34418200
H	2.68147700	-1.39012200	-1.34289400
H	3.06248900	-2.69001000	-0.21979700
H	1.75396200	-2.87746900	1.94976800

H	1.82778400	-1.16685200	2.39937700
H	0.26190400	-1.96491300	2.18381500
H	4.66695700	-0.69568900	-0.15420600
H	2.91461400	0.94437500	1.64238800
H	1.60404100	3.18521700	-0.71232000
H	2.07046000	3.01527300	1.00236500
H	3.18777100	3.71757900	-0.17463400
H	4.01584400	2.21271900	-2.11844900
H	3.94206000	0.45287100	-1.94552100
H	2.50570900	1.38066700	-2.42852000

TS_3b-4b

Sum of electronic and thermal Free Energies = -781.121706

Number of imaginary frequensies = 1

The magnitude of the imaginary frequency: -9.8116

C	0.07328200	-2.52476000	-0.45092900
C	-0.58801600	-1.24950100	-0.98078100
C	-1.99414400	-0.98009100	-0.92942500
C	-2.52827000	0.11265400	-1.77515200
C	-3.69699100	0.96600600	-1.26563900
C	-3.26059800	1.96697200	-0.18772900
C	-2.58296600	1.28615600	0.98515900
C	-1.08154000	1.45975200	1.15581900
C	-0.27983200	0.16557600	1.38162400
C	0.33925400	-0.40510300	0.12362300
C	1.19037200	-1.71165200	0.24743500
C	2.45135100	-1.63677700	-0.62850600
C	3.63901000	-0.87363800	-0.01196600
C	3.30203100	0.50217400	0.51430900
C	3.18176700	1.62474000	-0.20853800
C	2.83054100	2.93968700	0.43532900
C	3.36581700	1.67882200	-1.70211700
C	1.51085900	-2.19185400	1.65875200
C	-3.30942900	0.59435900	1.86966900
C	-2.89342100	-1.89953200	-0.20160300
H	-4.14795100	2.50760700	0.15507700
H	-2.87544600	-0.46001500	-2.65930200
H	4.05723700	-1.47113300	0.80417300
H	0.83528700	0.39830100	-0.42427100
H	-0.17863200	-0.91399300	-1.93872600
H	-0.54970900	-3.05758600	0.27250100
H	0.39621500	-3.22538500	-1.22632200
H	0.57924100	0.38352500	2.02873300
H	-0.87859900	-0.57727000	1.92176200
H	-0.92212800	2.12930100	2.00838700
H	-0.66629600	1.98760300	0.28888900
H	-2.86278000	0.10660600	2.73252300
H	-4.39107900	0.52359300	1.78015700
H	-2.59091600	2.71097000	-0.63227100
H	-1.70512300	0.74098100	-2.13491500
H	-4.49653100	0.32709300	-0.87889700
H	-4.11492600	1.50391800	-2.12099300
H	-2.83692400	-2.86892500	-0.72201000
H	-2.51695100	-2.08441700	0.80927700
H	-3.92570000	-1.56248600	-0.15429800
H	2.18838700	-1.17957300	-1.59415200
H	2.77110300	-2.66314800	-0.85000300
H	2.02395200	-3.15789700	1.60076000
H	2.16499000	-1.49898300	2.19420600
H	0.60601600	-2.33375400	2.25647800
H	4.42855200	-0.81158500	-0.76786000
H	3.14898600	0.58488800	1.59138800
H	2.67735500	2.83746700	1.51261200
H	3.62544100	3.67553000	0.27109000
H	1.91988400	3.36119100	-0.00920500
H	3.69807400	0.73052600	-2.12844500
H	2.42599000	1.96519100	-2.19317500
H	4.09851100	2.44791700	-1.96917100

TS_1-2

Sum of electronic and thermal Free Energies = -781.108302

Number of imaginary frequensies = 1

The magnitude of the imaginary frequency: -153.7101

C	0.00781700	-1.68773000	-0.26420100
C	-1.38680000	-1.48350400	-0.84207300
C	-2.55610100	-1.51647100	-0.17628400
C	-3.85805500	-1.46469800	-0.94433000
C	-4.94783700	-0.51649300	-0.41815000
C	-4.73638000	0.98288000	-0.69323700
C	-3.57225400	1.59637500	0.05312400
C	-2.25598000	1.70613400	-0.69671900
C	-1.03501900	1.37450600	0.18442600
C	-0.02837400	0.51105600	-0.41423500
C	0.86103200	-0.38554200	0.27162100
C	2.27460400	-0.44623500	-0.33295700
C	3.14481200	0.76721100	0.02591100
C	4.46652800	0.71591800	-0.69303800
C	5.61486300	0.22351700	-0.20831800
C	6.86924500	0.22097600	-1.04024600
C	5.77882700	-0.35875600	1.17057600
C	0.85275700	-0.31447100	1.79473400
C	-3.70964500	2.04797800	1.30272500
C	-2.66901500	-1.69991900	1.31141600
H	-5.65268600	1.50697600	-0.40548800
H	-4.26827300	-2.48372500	-0.91223500
H	2.61325200	1.68849200	-0.25768500
H	0.10760900	0.58698500	-1.49772500
H	-1.42666600	-1.38842800	-1.92665500
H	-0.01699500	-2.33096000	0.61888000
H	0.63889300	-2.14327300	-1.02862600

H	-0.41518400	2.27899300	0.37993800
H	-1.31636300	1.01815800	1.17978800
H	-2.16287700	2.71801300	-1.10551900
H	-2.27601300	1.02678100	-1.55378000
H	-2.89167600	2.49930700	1.85912700
H	-4.66926100	2.00358600	1.80971600
H	-4.61283100	1.13823300	-1.77250900
H	-3.65784700	-1.24866200	-2.00083400
H	-5.09107400	-0.66516500	0.65882800
H	-5.88912300	-0.80842100	-0.89433600
H	-3.48638900	-2.38849600	1.54763600
H	-1.75541200	-2.09288600	1.76291500
H	-2.91207200	-0.74701600	1.79845500
H	2.20120100	-0.53030700	-1.42488600
H	2.75802500	-1.36493600	0.02061200
H	1.52593200	-1.08013600	2.19048900
H	1.20421500	0.65865800	2.14929600
H	-0.14044300	-0.49364800	2.21345900
H	3.29279400	0.81577500	1.10832100
H	4.46349300	1.09635700	-1.71398500
H	7.24490000	-0.80006200	-1.16872400
H	6.70454200	0.65686400	-2.02768400
H	7.66028500	0.78950100	-0.53889100
H	6.51641200	0.21758200	1.73978600
H	4.85268600	-0.38968100	1.74792600
H	6.16679500	-1.38084900	1.09992000

TS_2-3

Sum of electronic and thermal Free Energies = -781.126377

Number of imaginary frequensies = 1

The magnitude of the imaginary frequency: -87.4146

C	-0.07125600	2.13551900	-0.07216000
C	0.99774400	1.32049300	-0.82804900
C	2.36890900	1.27688000	-0.44919800
C	3.37451100	0.89856100	-1.47437900
C	4.68054900	0.20841300	-1.06180700
C	4.53056800	-1.29215500	-0.76147300
C	3.51476200	-1.58998800	0.31934200
C	2.07848000	-1.83646300	-0.12195100
C	0.99300900	-1.06862900	0.66495600
C	0.24406000	-0.05892400	-0.17879800
C	-0.81130900	0.88460800	0.46057700
C	-2.18450300	0.69882000	-0.19549800
C	-2.86521100	-0.64216200	0.11223800
C	-4.11368800	-0.83174000	-0.71012200
C	-5.36146400	-0.51009700	-0.34467300
C	-6.52710700	-0.73776000	-1.27014700
C	-5.72865100	0.09236600	0.98604900
C	-0.90143500	0.83234800	1.98368000
C	3.87788900	-1.67313600	1.60216300
C	2.80782600	1.70886900	0.89372600
H	5.51172000	-1.67153800	-0.46245900
H	3.62272400	1.89811500	-1.88662700
H	-2.16830800	-1.46538200	-0.10281800
H	-0.13345100	-0.55751600	-1.07730400
H	0.86980200	1.27413000	-1.91016500
H	0.33173300	2.78727300	0.70650000
H	-0.66120000	2.74440800	-0.76119800
H	0.21742500	-1.75799500	1.02180800
H	1.41651400	-0.61230300	1.56543700
H	1.89140100	-2.91297000	-0.04337200
H	1.98038400	-1.61101600	-1.19107900
H	3.16427800	-1.89232200	2.39254500
H	4.91538400	-1.55295000	1.90286900
H	4.25646300	-1.81812300	-1.68419200
H	2.88176400	0.37475500	-2.30139500
H	5.12251300	0.71842900	-0.19970600
H	5.38866100	0.33307800	-1.88593200
H	3.20203500	2.73158600	0.76422200
H	1.99074500	1.76351900	1.61133200
H	3.61884800	1.09359900	1.28228300
H	-2.07562900	0.80203900	-1.28518200
H	-2.84129500	1.51956600	0.12295000
H	-1.62962400	1.57466200	2.32626200
H	-1.22693900	-0.14749900	2.34258500
H	0.05308300	1.06232400	2.46735800
H	-3.09442300	-0.70651200	1.17988800
H	-3.96559100	-1.24539400	-1.70756500
H	-6.21371500	-1.18164900	-2.21747600
H	-7.26213200	-1.40325600	-0.80369000
H	-7.04273200	0.20550000	-1.48222100
H	-4.86775000	0.29334800	1.62665500
H	-6.26369400	1.03641000	0.83405700
H	-6.41110100	-0.57126000	1.52876300
